# Design, development, and evaluation of the efficacy of a nucleic acid-free version of a bacterial ghost candidate vaccine against avian pathogenic *E. coli* (APEC) O78:K80 serotype

**DOI:** 10.1186/s13567-020-00867-w

**Published:** 2020-12-09

**Authors:** Safoura Soleymani, Amin Tavassoli, Gholamreza Hashemi Tabar, Gholam Ali Kalidari, Hesam Dehghani

**Affiliations:** 1 Division of Biotechnology, Faculty of Veterinary Medicine, Ferdowsi University of Mashhad, Mashhad, Iran; 2 Department of Clinical Sciences, Faculty of Veterinary Medicine, Ferdowsi University of Mashhad, Mashhad, Iran; 3 Department of Basic Sciences, Faculty of Veterinary Medicine, Ferdowsi University of Mashhad, Mashhad, Iran; 4 Stem Cell Biology and Regenerative Medicine Research Group, Research Institute of Biotechnology, Ferdowsi University of Mashhad, Mashhad, Iran

**Keywords:** avian colibacillosis, *E. coli*, O78:K80 serotype, bacterial ghosts technology, vaccine, nucleic-acid free

## Abstract

One of the major bacterial infectious diseases in the poultry industry is avian pathogenic *Escherichia coli* (APEC), which causes colibacillosis in chickens. To develop a novel nucleic acid-free bacterial ghost (BG) vaccine against the O78:K80 serotype of APEC, in this study we constructed a plasmid that harbored E-lysis and S nuclease (SNUC). Following the expression, the O78:K80 bacteria lost all of their cytoplasmic content and nucleic acids by enzymatic digestion. The functionality of these two proteins in the production procedure of bacterial ghosts was confirmed by monitoring the number of colonies, scanning electron microscopy imaging, gel electrophoresis of genomic DNA, and qPCR on the plasmid content of bacterial ghosts. The protective efficacy of the ghost vaccine generated from O78:K80 serotype of APEC was tested in chickens by injection and inhalation routes and compared with that in chickens that received the injection of a killed vaccine. The O78:K80 BG vaccine candidate, used as injection and inhalation, in comparison with the killed vaccine, triggered higher proinflammatory cytokine expression including IL-6, IL-1β, and TNFSF15; a higher level of antibody-dependent humoral (IgY and IgA) and cellular immune responses (IFNγ and lymphocyte proliferation); and lower lesion scores. According to the results of this study, we suggest that the bacterial ghost technology has the potential to be applied for the development of novel vaccines against avian colibacillosis. This technology provides an effective and reliable approach to make multivalent vaccines for more prevalent APEC strains involved in the establishment of this infectious disease in the poultry industry.

## Introduction

Avian colibacillosis is one of the most commonly occurring bacterial diseases caused by avian pathogenic *Escherichia coli* (APEC) [1]. It is a localized or systemic infection that manifests with various signs including, acute fatal septicemia, subacute pericarditis, airsacculitis, and perihepatitis. This infectious disease is generally associated with respiratory disease in poultry flocks, which in severe cases, leads to septicemia and is responsible for a significant proportion of the mortality [1–4]. The mortality rate in chickens is 1–10%, although, for broilers and commercial organic chicken, it could be higher [5]. The effects of this disease on poultry production and slower growth rate is economically devastating worldwide [2, 6, 7]. The avian colibacillosis is frequently associated with O78:K80, O1:K1, and O2:K1 serotypes [3, 4]. Therefore, preventive measures that would protect against these three serotypes of avian colibacillosis have the potential to prevent a large number of APEC outbreaks [2].

As a result of increasing demands for poultry production, the threat of APEC is higher than ever today [1]. There have been numerous attempts to control and treat the affected birds. Antibiotics play a significant role in the control of APEC infections, but their continuous use may cause the emergence of drug resistance and or multidrug-resistant strains [8–10]. In addition to antibiotics, vaccination is considered an important preventative tool for controlling the APEC outbreaks and, more importantly, reducing antibiotic usage. Many efforts have been made to develop a variety of vaccines and vaccination routes against APEC, including passive immunization; use of killed, live, subunit, and recombinant vaccines; and vaccines based on specific virulence factors. Passive immunization is efficient only for 2 weeks in young chickens [2, 11–13]. Killed vaccines are known as the ‘emergency’ vaccine in response to a colibacillosis outbreak against the strains isolated from the affected birds. The live attenuated *E. coli* vaccine similar to passive immunization and killed vaccines leads to protection against homologous strains. These vaccination strategies are less efficient against heterologous strains. Experimental evidence indicates that the subunit, multi-antigenic, and genetically modified candidate vaccines are more successful to stimulate broad protection, especially in chickens challenged with heterologous strains [2, 14, 15]. Since multiple strains of APEC are involved in the outbreaks, the development of more promising vaccines with sufficient protection is very important. Therefore, introducing a multivalent vaccine, with the ability to protect against several strains of colibacillosis with safe administration to chickens, could reduce the severity of APEC infections [2, 14].

Bacterial ghosts (BGs) are cell envelopes that are derived from Gram-negative bacteria, lacking cytoplasmic contents while preserving the cellular morphology including all cell surface structures [16, 17]. BGs have been produced by controlled expression of the cloned lysis gene E of PhiX174 bacteriophage. The expression of the E-lysis protein leads to the formation of a trans-membrane tunnel structure through the inner and outer bacterial membranes. By the formation of those tunnels, all of the cytoplasmatic contents are expelled into the surrounding medium, resulting in ghosts that are pure envelopes [16–19]. The bacterial ghost system has been proposed as a novel vaccine candidate and drug or active substance delivery vehicle, usually in that it combines excellent natural intrinsic adjuvant properties with versatile carrier functions for foreign antigens [16, 20, 21]. Furthermore, BGs having the intrinsic adjuvant properties exhibit efficient tropism to the host’s antigen-presenting cells (APCs) and trigger a potent humoral and cellular immune response. Multiple antigens of the native envelope of BGs and recombinant protein or DNA antigens can be combined in a single type of BG. Antigens can be presented on the inner or outer membrane of the BGs as well as in the periplasm that is sealed during BG formation. Antigenic epitopes can be inserted into flagella, fimbriae, or proteins of the outer membrane or periplasm. Further advantages of BG vaccines include the simplicity of the production method, safety, long shelf life without the need for cold chain storage, needle-free administration, and versatility as a combination vaccine [20–25].

Despite all these remarkable advantages and benefits of BGs, there is a concern about the possibility of the presence of viable and reproductive bacterial cells at the end of the BG production process [17, 25]. To guarantee the total inactivation of target bacteria in BG preparation, the expression of a secondary lethal gene has been applied. For this purpose, the intracellular expression of staphylococcal nuclease (SNUC) without the signal sequences for extracellular release has been used [17, 26]. The expression of this enzyme at the presence of Ca^2+^ and Mg^2+^ resulted in cleaving either single- or double-stranded DNA or RNA to mono, di, or oligonucleotides. This intracellular degradation restricts or abolishes the reproductive cells in lysis media [17, 25]. Importantly, the generation of the nucleic acid-free version of BGs eliminates the risk of antibiotic resistance gene transfer to hosts. Due to the presence of E-lysis and SNUC genes and also all the mentioned properties and advantages, BGs turn out to be a desired therapeutic agent, which is especially applicable for gram-negative bacterial diseases [17, 25, 26].

In our previous studies, the O78:K80 and O2:K1 BGs were produced using a construct with the PhiX174 lysis gene [27, 28], and the chicken host immune responses were evaluated with several limited tests. In this project, we constructed a nucleic acid-deficient version of *E. coli* O78:K80 bacterial ghost using a plasmid containing the PhiX174 lysis and SNUC genes. Here for the first time, we have set up a procedure for the preparation of the O78:K80 strain at the fermentor scale. We have also characterized the lysis and SNUC activity using specific techniques. The prepared O78:K80 BG candidate vaccine without any supplement or adjuvant has been used with doses higher than the previous trial [27]. The BG candidate vaccine was tested as an inhalation and injection vaccine in host chickens, which were then challenged with a single dose of wild type O78:K80. The humoral and cellular immune responses, the expression of proinflammatory cytokines, and the lesion scores were compared to those in chickens that received the killed bacteria supplemented with adjuvant and were challenged. We demonstrate that the non-living O78:K80 BGs are equally effective as the killed bacteria in eliciting significant protection. Since simultaneous infection with different strains of avian pathogenic *E. coli* is considered a major public health burden, developing a multivalent BG vaccine using the more prevalent strains of APEC would greatly reduce the hazards. The methodology used to develop a BG candidate vaccine against O78:K80 in this study is scalable and can be applied for producing multivalent BG vaccines against main avian colibacillosis serotypes.

## Materials and methods

### Generation of constructs and production of bacterial ghosts

**Figure 1:**
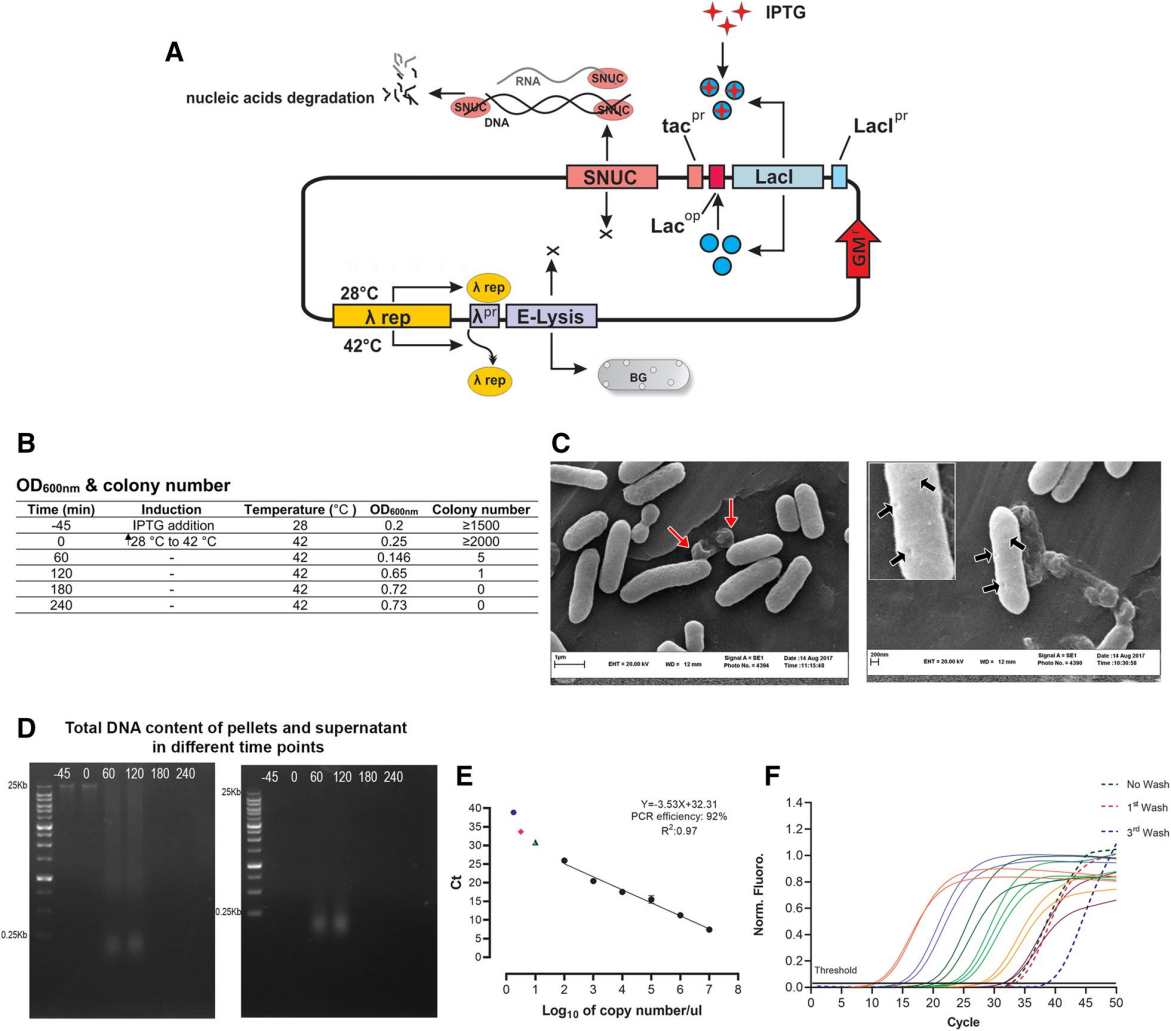
**Procedure and quality control of ghost production from** ***E. coli*** **O78:K80.** The schematic representation of the *E. coli* O78:K80 bacterial ghost production procedure. **A** The pmET32c vector (Genebank: MN049497.1) contains the gentamicin-resistant, E lysis, and SNUC genes; the expression of the latter two genes is under the control of temperature-sensitive lambda-repressor CI857 (λ rep) and LacI inhibitors. The expression of these two genes is induced by increasing temperature from 28 to 42 °C and the addition of IPTG. Following the expression of E-lysis and SNUC (S nuclease), the *E. coli* O78:K80 is turned to the bacterial ghost which loses all cytoplasmic contents through the cell wall pores, and the DNA and RNA contents by enzymatic digestion. The other regulatory elements shown in the pmET32c vector include the LacI promoter (LacI^pr^), Lac operator (Lac^op^), and tac promoter (tac^pr^). The functionality of E-lysis and SNUC proteins in bacterial ghost production steps were checked and confirmed as shown in **B**–**F**. **B** Growth kinetics of *E. coli* O78:K80 harboring pmET32c vector was monitored by measuring the optical density of bacteria at 600 nm (OD_600 nm_) and colony number determination at different time points during the BG generation procedure. The OD_600 nm_ reading shows a drop 60 min after E-lysis induction (28 °C to 42 °C) and increases as a result of the release of bacterial cell cytoplasmic contents. The time points are based on the minutes relative to E-lysis induction (0 min). **C** Scanning electron microscopy (SEM) of *E. coli* O78:K80 ghosts before (left panel) and at the end of washing steps (right panel). Black arrows in the right panel and inset indicate the lysis-induced pores in BGs and red arrows in the left panel show the release of cytoplasmic contents through these pores. **D** The expression of SNUC results in the degradation of nucleic acids. The total DNA content at different time points of BG production steps within pellets (left panel) and the supernatants (right panel) were visualized by 0.7% agarose gels electrophoresis. **E**, **F** Quantitative analysis of pmET32c vector content of BGs before and after 3 rounds of washing via absolute quantitative real-time PCR based on the measurement of copy number. The amplification and standard curves of pmET32c vector copy numbers are shown. It is illustrated that at the end of the BG production process and after the first and third washings of *E. coli* O78:K80 ghosts, the vector copy numbers fell out of detection limits below 100 copies. No washing: green triangle; After first washing: red square; and after third washing: blue dots.

The pmET32b plasmid (GenBank: KF561236.1) [27, 28] containing the E-lysis gene, lambda promoter (λ^pr^), temperature-sensitive lambda-repressor CI857 (λ rep) and gentamicin resistance gene (GM^r^) was used for subcloning of the staphylococcal nuclease *A* gene. For this purpose, we designed an expression cassette that contained LacI and its promoter (LacI^pr^), Lac operator (Lac^op^), tac promoter (tac^pr^), and the mRNA sequence of nuclease *A* of *Staphylococcus aureus* (Genbank: V01281.1) without its signal sequence and fused to TrxA and the 6×His affinity tag. This DNA fragment (2640 bp) was chemically synthesized (Shinegene CO., Shanghai, China). This fragment was digested with SspI and AdeI and was inserted into the corresponding sites of pmET32b plasmid and transformed in DH5α competent cells. Transformed cells were cultured in an LB plate supplemented with gentamicin (10 µg/mL) and 1% glucose and incubated at 28 °C overnight. The correct bacterial colonies were confirmed with colony PCR by SNUC-F and SNUC-R primers (Table 1). This plasmid (here after named pmET32c; Genebank: MN049497.1) containing both E-Lysis and SNUC genes (Figure 1A) was confirmed by restriction digestion, and sequencing using the above primers (Macrogen Inc., Seoul, South Korea) (Additional file 1).

**Table 1:** List of oligonucleotides used in this study

Name	Sequence (5′–3′)	Product (bp)	Annealing T (°C)	Application
SNUC-F	ACAATTCGTTCAAGCCGAG	513	58	Colony PCR confirmation
SNUC-R	AATGTAATTCAGCTCCGCC			
pmET32c-F	CATGCATTGACAATTAATCATC	110	52	qPCR to evaluate the plasmid (MN049497.1) content of BGs
pmET32c-R	ACCTCCTCTCCTTCTTAA			
ACTB-F	CGTGACATCAAGGAGAAG			RT-qPCR for β-actin (NM_205518)
ACTB-R	AGGACTCCATACCCAAGAAAGATG	187	57	
Probe	FAM-CACCTGAACCTCTCATTGCCAA-BHQ1			
IL1b-F	CTTCGACATCAACCAGAA			RT-qPCR for IL1b (NM_204524)
IL1b-R	CGACATGTAGAGCTTGTA	196	55	
Probe	FAM-TGCTTCGTGCTGGAGTCACC-BHQ1			
IL6-F	CTGGAATTCATTCAAGAGA			RT-qPCR for IL6 (NM_204628)
IL6-R	CATCGGGATTTATCACCATCTG	112	57	
Probe	FAM-AACGTCGAGTCTCTGTGCTACA-BHQ1			
TNFSF15-F	GAGCACACCTGACAGTGAAGAA			RT-qPCR for TNFSF15 (NM_001024578)
TNFSF15-R	CTCGGAAAGTGACCTGAGCAT	183	64	
Probe	FAM-CCTCCAGCACCACGGGAAGCCATCTG-BHQ1

The wild type avian pathogenic *E. coli* O78:K80, strain χ1378 (a gift from the Microbiology Division, Faculty of Veterinary Medicine, Tehran University, Tehran, Iran [29]) was transformed with pmET32c plasmid and was used for the production of bacterial ghosts. To generate the nucleic acid-free version of *E. coli* O78:K80 BGs, the expression of the E-lysis and SNUC genes were triggered by thermal inactivation of λ repressor at 42 °C and the addition of IPTG to the culture medium, respectively. We set up growth conditions for the production of BGs in both the small- and large-scale cultures.

In the small-scale culture, a single transformed colony was added to the SOB^++^ medium (pH 7.0 and supplemented with 10 µg/mL gentamicin and 1% glucose) and cultured overnight in a shaker incubator at 28 °C temperature with an aeration rate of 120 rpm. Then, 0.5 mL of this overnight culture was used to inoculate 100 mL of SOB^++^ medium (containing 10 µg/mL gentamicin), which was incubated at 28 °C with shaking (150 rpm) to reach an optical density at 600 nm (OD_600 nm_). At an OD_600 nm_ of 0.2–0.3, to induce the SNUC expression, IPTG (to a final concentration of 5 mM) was added to the culture medium. After 45 min, the E-lysis expression was induced by increasing the temperature from 28 to 42 °C. The induction time points for SNUC and E-lysis proteins were considered − 45 min and 0, respectively. Ninety minutes after the start of E-lysis induction, MgCl_2_ and CaCl_2_ were added to a final concentration of 1 mM and 10 mM, respectively, to stimulate nucleic acid digestion activity of SNUC. OD_600 nm_ was monitored for 4 more hours at 1-h time points.

In the large-scale culture, all conditions were exactly similar to those for the small-scale culture except for the IPTG which was used at a 2.5 mM concentration.The O78:K80 BGs were cultured in 2 L of the SOB^++^ medium in a 5 L fermentor with a stirring rate of 300 rpm without adding any antifoam. The pH was stably maintained between 6.5 to 7.5 by the continuous addition of NaOH (1M) and HCl (1N) during the BG production procedure. At − 45 min, 0, 1, to 4 h time-points in the BG generation process, sampling was carried out to evaluate the accuracy and efficiency of the procedure. At the end of the procedure, the O78:K80 BGs were collected by centrifugation and washed three times with sterile 0.9% NaCl solution. Then, the O78:K80 BGs were diluted in NaCl (0.9%) and stored at − 80 °C for further applications.

### Evaluation of the efficiency of lysis process and nucleic acid degradation

Multiple tests were performed to confirm the functionality of E-Lysis and SNUC genes for the generation of BGs and digestion of bacterial nucleic acids. For monitoring the growth and lysis of the O78:K80 bacteria, as mentioned in the “Generation of constructs and production of bacterial ghosts” section, in − 45, 0, 1 to 4 h after E-lysis induction, the samples were subjected to the OD_600 nm_ measurement. To determine the number of colonies, the bacterial samples were diluted with NaCl (0.9%). Then, 100 µL of the diluted samples (1:1000 dilution) were cultured in LB agar plates overnight at 28 °C.

To analyze the efficiency of nucleic acid digestion by SNUC, the DNA content of the pellet and supernatant was extracted from 1 mL of samples taken at different time points during BG production. The bacterial cells were harvested by centrifugation at 4000 rpm for 5 min. DNA content of both the supernatant and pellet (from 500 µL of culture) was extracted using the Genomic DNA Isolation Kit (DENAzist Asia Co., Mashhad, Iran). The extracted DNA contents were eluted in 100 µL of elution buffer and were subjected to electrophoresis in 0.7% agarose gels.

To evaluate the plasmid content of BGs, three sets of samples were prepared at the end of the BG production process from 10^11^ BGs/mL, before the first wash, and before the third washing steps. These samples were subjected to plasmid extraction (NanoPlus plasmid extraction kits, Bioneer Inc., Daejeon, South Korea) and real-time PCR assay with SYBR-Green (RealQ Plus 2× Master Mix Green, AMPIQON, Odense, Denmark) by specific forward and reverse primers (pmET32c-F and pmET32c-R; Table 1, Additional file 2). The PCR program contained an initial denaturation at 94 °C (5 min), followed by 50 cycles of 95 °C for 30 s, 52 °C for 30 s, and 72 °C for 30 s. The melting analysis was performed between 60 and 98 °C in steps of 1 °C (Additional file 2). To calculate the exact copy number of the likely undigested plasmids, we generated a calibration curve with seven dilutions (10^−1^ to 10^−7^) from 10^10^ copies/µL pmET32c plasmid stock solution.

The surface morphological features of O78:K80 cells in bacterial ghost preparation were analyzed by Scanning Electron Microscopy (SEM). The sampling was done in two stages: before and after washing steps. The samples were prepared based on the protocol described by Witte et al. [30] with some differences. Briefly, at first, the BGs were fixed (2.5% glutaraldehyde, 60 min), washed, and post-fixed (1.5% osmium tetroxide, 15 min). Then, the dehydration step was performed using serial dilutions of ethanol (30%, 50%, 70%, 90%, and 100%). Finally, the samples were coated with gold–palladium alloy and imaged by scanning electron microscope (LEO-1450, Zeiss, Oberkochen, Germany).

### Vaccination and challenge scheme

The Ross 308 broiler chickens (provided by Seamorgh chicken farm, Mashhad, Iran) were randomly divided into five groups (at least 25 chickens in every group). Each group of birds was housed in individual pens with a distance of at least 1.5 m from the other pens under constant room temperature, regular dark/light period, and regular feeding. The study was performed in the Chicken experimental housing of the Faculty of Veterinary Medicine, Ferdowsi University of Mashhad. The first group of chickens (Inj. BGs + Expo.) was injected (subcutaneously in the caudal abdominal area) with 1 mL (10^11^ BGs/mL) of *E. coli* O78:K80 BGs. In the second group (Inhal. BGs + Expo.), immunization of chickens was performed by the inhalation of BGs. For this purpose, 100 mL of 10^11^ BGs/mL suspension was sprayed exactly from the top into the enclosed pen. In the killed vaccine group (the third group; killed + Expo.), vaccination was performed with 1 mL of 3 × 10^9^ CFU/mL of *E. coli* O78:K80. Chickens in these groups were vaccinated on days 7, 14 and 21, and then were subjected to the injection of 1 mL of 1.2 × 10^9^ CFU/mL of wild type *E. coli* O78:K80 on day 28. Before exposure, the immune system of chickens was suppressed by injection of dexamethasone (1 mg/kg) and administration of the eye drop vaccine of infectious bronchitis (IB) H120 (dose 10^3^ EID_50_) on days 25, 26 and 27. In the non-vaccinated/only exposure group (only Expo.; the group 4), chickens did not receive any form of the vaccine but challenged exactly in a similar manner to the vaccinated groups. In the fifth group (Neg. Ctrl), chickens were neither immunized nor challenged. Chickens in all groups were monitored every day for any clinical symptoms, lesions, and mortality until day 38. All experiments were performed based on the ethical guidelines of the Committee on Research Ethics of the Ferdowsi University of Mashhad, Mashhad, Iran.

For the preparation of the inactivated *E. coli* O78:K80 to be used as the killed vaccine, 3% formaldehyde was added to the 3 × 10^9^ CFU/mL of bacterial cells with adjuvant and incubated at 37 °C for 5 h with continuous shaking. A mix of 0.1% ALK(SO_4_)_2_ and 0.37% KOH were used as the adjuvant in the first vaccination. In the second and third vaccinations, the concentration of this adjuvant was doubled. To check the viability of bacterial cells in the killed vaccine, the final suspension was cultured on the LB agar plate.

### Immune response analysis experiments

Sera were prepared from chicken’s blood samples at days 14, 21, 28, and 38 and stored at − 80 °C until they were used for IgY assay. To obtain tracheal lavage samples, chickens were euthanized by decapitation at desired times. The tracheal lavage fluids were obtained by five times sequential washings of the lungs with a single volume of PBS (1 mL, 1×). Samples were stored at − 80 °C for IgA antibody detection. Antibodies against IgY (A00165, goat anti-chicken IgY antibody [HRP], pAb, GenScript Co., Piscataway, USA) and IgA (MBS560178, goat anti-chicken IgA polyclonal antibody, [HRP], MyBioSource) were used to determine the titers in 1:10 diluted serum samples and tracheal lavages by indirect enzyme-linked immunosorbent assay (ELISA) based on the recommended protocol (GenScript Co., Piscataway, USA) with some adjustments.

The presence of IFNγ in 1:10 diluted serum samples was monitored at days 14, 21, 28, and 38 with a direct sandwich ELISA. The anti-chicken IFN-γ (Rockland Immunochemicals Inc., Limerick, USA) and HRP-conjugated rabbit anti-chicken IFN-γ polyclonal (CUSABIO Technology LLC., Houston, USA) antibodies were used for assessment of IFNγ levels.

The lymphocyte proliferation analysis in immunized and non-immunized chickens was performed on days 21, 28, and 38. Lymphocytes were isolated from spleen tissues. Briefly, after sacrificing chickens, the spleen tissues were collected aseptically and placed in sterile cold PBS (1×). Single-cell suspensions were prepared by gently pushing the splenic pulp through a sterile nylon mesh with a pore size of 70 µM. Cells were washed and resuspended in 3 mL of cold PBS (1×) and then layered over 3 mL of Lympholyte^®^-H (CEDARLANE Co., Burlington, Canada). The preparations were enriched for lymphocytes by centrifugation (at 2000 rpm) for 30 min. Cells were recovered from the interface, resuspended in cold PBS (1×), and washed twice in 3 mL of cold PBS (1×). Then, the collected lymphocytes were diluted in prewarmed cell culture medium at 10^5^ per 100 µL and added to wells of 96-well plate with three replicates for each sample. To each well, 12.5 µg/mL Concanavalin A (Con A) was added as a stimulator. The plate was incubated in a CO_2_ incubator at 41 °C for 48 h. AlamarBlue (AB) was added to each well with a dilution of 1:10 and mixed gently by pipetting. After 4 h, the OD was read at 570 nm (low wavelength) and 600 nm (high wavelength). The absorbance of AB in the culture medium and medium alone (without AB) was measured at both 570 nm and 600 nm. The absorbance of the medium alone (without AB) was subtracted from the absorbance of the medium containing AB at both low and high wavelengths (AO_LW_ and AO_HW_, respectively). Then, the correction factor R0 was calculated as AO_LW_/AO_HW_. The percent of proliferative cells was determined by “AO_LW_ − (AO_HW_ × R0) × 100” formula. Proliferative cells reduce a higher percentage of AB than non-proliferating cells. The means of AO_LW_ and AO_HW_ in triplicate were considered for each group of experiments at each time point.

At 10 days post-infection (38 day-old chicken), peripheral blood mononuclear cells (PBMCs) were isolated from the whole blood of at least three chickens in each group. Briefly, the whole blood was collected in a tube containing K-EDTA, mixed thoroughly, diluted 1:1 with PBS (1×), and layered over Lympholyte^®^-H (CEDARLANE Co., Burlington, Canada). Then the tubes were centrifuged at 2000 rpm for 30 min with no brake. PBMCs were removed from the interface and washed twice in PBS (1×). Then, they were resuspended in the appropriate medium, counted, and their number was adjusted to 1 × 10^6^ cells of PBMCs/mL and stored at − 80 °C for evaluating cytokine production via RT-qPCR.

Total RNA of PBMCs were obtained by Total RNA Isolation Kit (DENAzist Asia Co., Iran). The quality and quantity of extracted RNAs were evaluated using gel electrophoresis and Nanodrop spectrophotometry. Total RNAs (4 μg) were reverse transcribed using random hexamer primers and MMLV reverse transcriptase (Thermo Fisher Scientific, Waltham, MA, USA).

To quantify the level of transcripts for β-actin, IL-6, IL-1β, and TNFSF15 in each group, three quantitative RT-PCR reactions containing RealQ Plus 2× Master Mix for Probe, 2 μL cDNA template, forward and reverse primers (500 nmol), and 5′FAM-3′BHQ1-labeled probe (100 nmol) in a 20 μL reaction volume, were performed in a Rotor-Gene Q real-time PCR cycler (Qiagen, Germantown, USA). The sequences of primers and probes used for RT-qPCR are shown in Table 1. Amplifications steps were 95 °C for 15 min, followed by 40 cycles of 95 °C for 30 s, annealing temperature for each gene (Table 1) for 30 s, and 72 °C for 30 s. Gel electrophoresis was performed to confirm the identity of PCR products (Additional file 3). Data were calculated using the copy number of target transcript and reference gene (β-actin). The serial dilutions of amplified fragments were used to make the standard curves. For each dilution, the real-time reading was done in triplicate and the log of copy numbers was plotted against the cycle threshold (Ct). The efficiency (E) for each qPCR reaction was calculated based on the E = (10^−1/slope−1^) × 100% equation using the slope of standard curves. The correlation coefficient (R^2^) was acceptable in the standard curve of all four genes (Additional file 3). Absolute copy numbers for IL-6, IL-1β, TNFSF15, and β-actin transcripts were quantified based on the related standard curves. For each cDNA, the quantity of the target transcript (IL-6, IL-1β, TNFSF15) was divided by the quantity of the reference gene (β-actin) and plotted.

### Evaluation of necropsy lesions in liver, heart, and air sacs

The scoring assessment of necropsy lesions in the liver (perihepatic lesions), heart (pericardial lesions), and air sacs was performed 10 days after exposure with wild type O78:K80 on day 38. All chickens were euthanized by decapitation in a sterile condition. Scoring for air sac necropsy assessment was according to Kleven et al. [31] as follows: 0: no lesions, 1: cloudiness of air sacs, 2: thickened air sac membranes, 3: “meaty” appearance of membranes with large accumulations of a cheesy exudate in one air sac, and 4: lesions similar to that of score 3 but in two or more air sacs. The pericardial and perihepatic lesions were scored according to Charleston et al. [32]. Briefly, for perihepatic lesions, 0 indicates no visible lesions; 1 indicates definite fibrination on the surface of the liver; and 2 indicates extensive fibrination, adhesions, liver swelling, and necrosis. In the pericardial lesions, scoring was as follows: 0: no visible lesions, 1: excessive clear or cloudy fluid in the pericardium, and 2: extensive fibrination in the pericardial cavity. Representative images for each score in the air sacs, liver, and heart lesions are shown in Additional file 4.

### Statistical analysis

For antibody assays, assessment of cellular immune response (IFNγ), and necropsy lesion scoring samples were collected from at least five chickens at every desired time point. For lymphocyte cell proliferation assay samples were collected from at least three chickens per group at 21, 28 and 38 days, and for RT-qPCR analysis of proinflammatory cytokines, samples were collected from at least three chickens per group at 10 days post-infection (38 day-old chicken). Statistical analyses were performed using GraphPad Prism software version 8.0. Kruskal–Wallis test followed by Dunn’s multiple comparisons analysis were used to find statistically significant differences. Differences were considered significant when the *p* value was ≤ 0.05. The significant differences between groups are shown as ****p ≤ 0.0001, ***p ≤ 0.001, **p ≤ 0.01 and *p ≤ 0.05.

## Results

### Generation of nucleic acid-free bacterial ghost against O78:K80

Production of *E. coli* O78:K80 ghosts was induced by activating the expression of the E-Lysis gene in the pmET32c vector (Figure 1A). The intracellular accumulation of SNUC is under the control of the tac promoter, which is induced by the addition of IPTG. The OD_600 nm_ of this bacterial culture decreased 60 min after E-Lysis induction due to O78:K80 cell lysis. Then, this optical density was increased as a result of cytoplasmic expelling to the medium and remained constant for the next 4 h (240 min) when the BGs were harvested (Figure 1B). The number of viable cells was also decreased after the expression of E-Lysis and SNUC. As shown in Figure 1B and Additional file 5, the colony number at different time points (from the start of induction until the time of BG harvest) reached zero, indicating the absence of viable cells.

The SEM imaging was used to confirm the formation of pores by the E-Lysis protein in the O78:K80 bacterial cell envelope. The expelling of cytoplasmatic contents through these pores into the surrounding medium was recorded during the BG generation (Figure 1C, left panel). Pores were observed in the pole and at the center of dividing cells (Figure 1C, right panel).

To determine the intracellular effect of the SNUC on the nucleic-acid digestion of bacterial cells during the BG production procedure, the electrophoretic analysis of bacterial ghosts’ genetic content was performed. For this purpose, the total DNA was extracted from the pellet and supernatant of samples at different time points. Before the start of E-Lysis action (− 45 and 0 min), the undigested DNA of bacterial cells around 25 kbp in size was observed (Figure 1D). However, by nuclease expression in the first 2 h of SNUC induction (60 and 120 min), the bacterial cell genomic DNA has rapidly degraded to oligonucleotides shorter than 100 bp in the supernatant (Figure 1D, left panel) and the pellet (Figure 1D, right panel). There were no detectable genomic DNA or short oligonucleotides with longer inductions (180 and 240 min, Figure 1D).

To investigate the plasmid DNA content inside the ghosts at the end of the production process and washing steps, the extracted pmET32c plasmid samples were subjected to absolute qPCR by specific primers. The standard curve with a linear dynamic range from 10^2^ to 10^7^ copies (plasmids)/µL was prepared (Figure 1E, F) and used for quantification. We found the copies of extracted pmET32c plasmids undetectable after one and three washing steps, indicating the low copy number of fewer than 100 copies which was out of the limit of detection in the standard curve. These findings showed that the produced O78:K80 BGs had negligible copies of plasmid, thus partially eliminating the concerns about the safety of BG vaccines.

### Humoral immune responses

**Figure 2:**
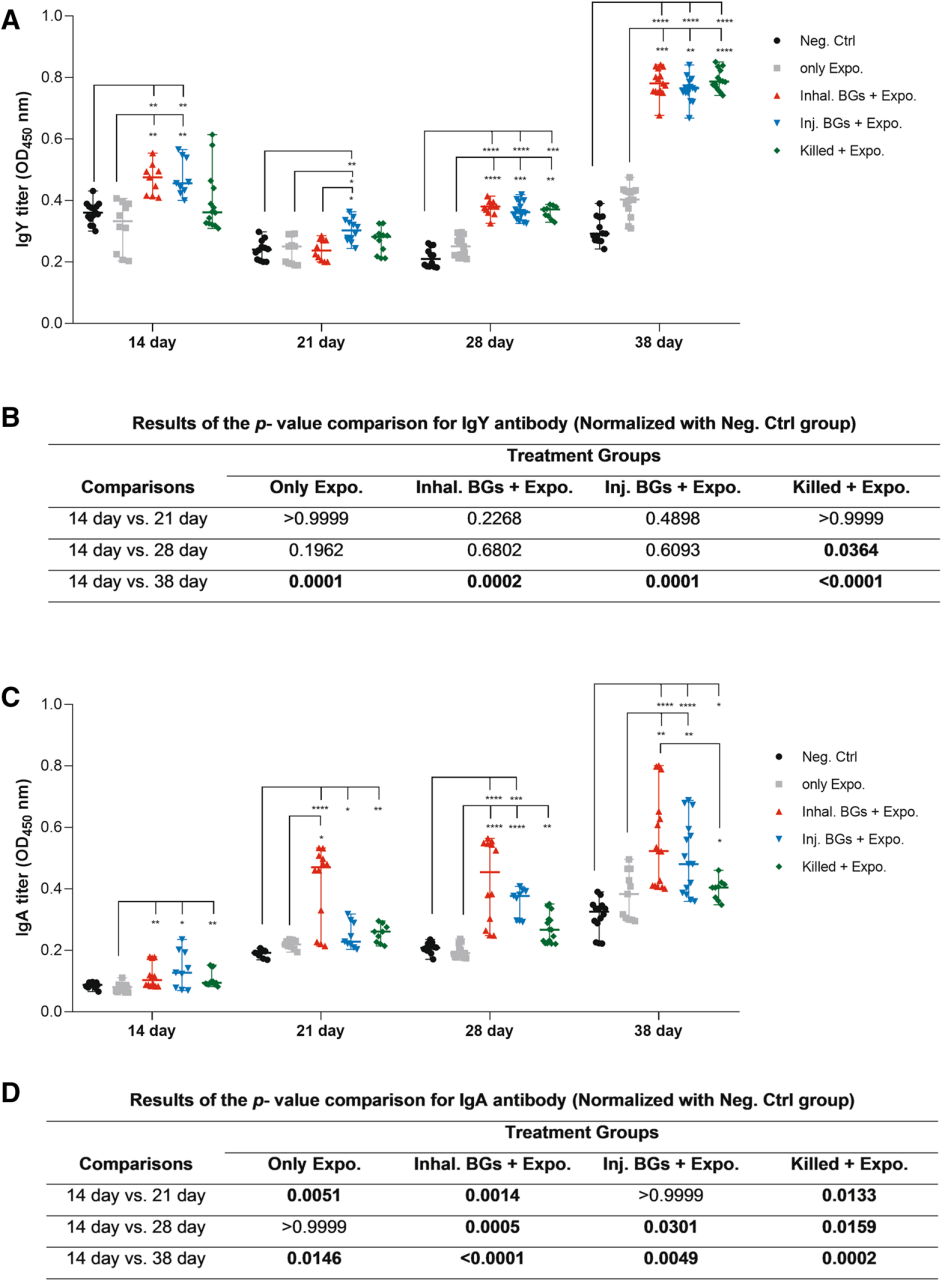
**Humoral immune responses.** **A** IgY titers of sera from different groups on days 14, 21, 28, and 38. The serum samples were diluted 1:10 before the ELISA assay. **B** The *p* values for pairwise comparisons of IgY titers between different groups are shown. **C** Tracheal lavage IgA titers of different groups on days 14, 21, 28, and 38. The lavage samples were diluted 1:10 before the ELISA assay. **D** The *p* values for pairwise comparisons between different groups for IgA antibodies. Chickens were immunized with BGs through inhalation (Inhal. BGs + Expo.), BGs by injection (Inj. BGs + Expo.), and injection of killed vaccine (killed + Expo.) on days 7, 14, 21, and were challenged on day 28 with the wild type *E. coli* O78:K80. Chickens in the only exposure (only Expo.) group without immunization were challenged. Chickens in the negative control (Neg. Ctrl.) group neither received any immunization nor challenged. Data represent the OD_450 nm_ of at least three to five chicken samples in triplicate. Data in both **B** and **D** were normalized with the corresponding data in the Neg. Ctrl group. The bold font in **B** and **D** represents the statistically significant *p* values (*p* < 0.05).

Titers of IgY and IgA antibodies against O78:K80 bacteria in the serum and tracheal lavage fluid samples were analyzed by indirect ELISA on days 14, 21, 28, and 38. We found that the birds immunized through inhalation and injection of BGs had significantly higher titers of IgY and IgA than that of non-immunized birds in the Neg. Ctrl. and Only Expo. groups (*p* < 0.001) after the first vaccination on day 14 (Figure 2). However, on day 21, the highest IgY titers belonged to the Inhal. BGs + Expo group. At days 28 and 38, the IgY titers in three vaccinated groups were significantly higher than those of the birds in the Neg. Ctrl. and Only Expo. groups (*p* < 0.05). Also, there were no significant differences in the IgY titers between birds in the three immunized groups. Sequential monitoring of IgY titers from day 21 toward day 38 showed an increasing trend in the immunized groups. We observed a decrease in IgY levels from days 14 to 21, which could be attributed to the reduction of maternal antibodies (Figure 2A). Normalizing the IgY data in four experimental groups with the Neg. Ctrl. group at every time point showed that there were significant differences between the titers of each time point versus that of day 14 (Figure 2B and Additional file 6).

The titers of IgA antibody in the immunized chickens were significantly higher than those of non-immunized chickens after one, two- and three-times vaccination at days 14, 21, and 28 (*p* < 0.05). The highest IgA titers at these time points belonged to the chickens immunized with BGs through inhalation. We detected the highest IgA titers on day 38 in all three vaccinated groups, which were significantly higher than those of the Neg. Ctrl and Only Expo. groups (*p* < 0.05). Moreover, it was observed that the antibody titers after exposure were higher in the two vaccinated groups with BGs than the titers of the killed vaccine group. The IgA titers in four experimental groups were normalized with the Neg. Ctrl. group at every time point and pairwise comparisons were made for each time point versus that of day 14 (Figure 2D and Additional file 6). The results of these comparisons demonstrated that the IgA levels in days 28 and 38 were significantly higher.

### Cellular immune responses

**Figure 3:**
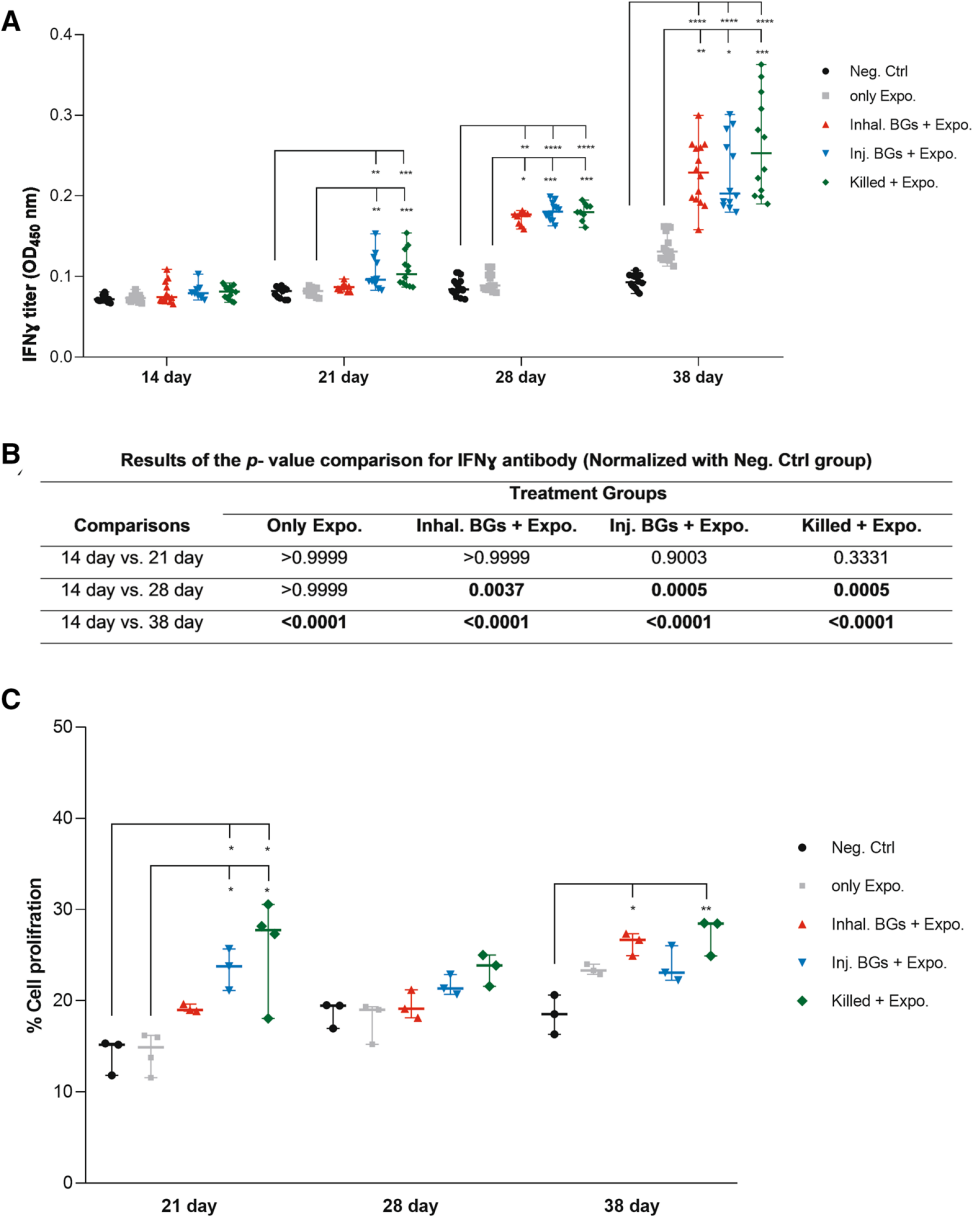
**Cellular immune responses.** **A** The IFNɣ titers of different groups on days 14, 21, 28, and 38. The serum samples were diluted 1:10 before the ELISA assay. **B** The *p* values for pairwise comparisons between different groups for IFNγ titers. **C** Percentage of spleen cell proliferation in different groups on days 21, 28, and 38. Chickens were immunized with BGs through inhalation (Inhal. BGs + Expo.), BGs by injection (Inj. BGs + Expo.), and injection of killed vaccine (Killed + Expo.) on days 7, 14, 21, and were challenged on day 28 with the wild type *E. coli* O78:K80. Chickens in the only exposure (only Expo.) group without immunization were challenged. Chickens in the negative control (Neg. Ctrl.) group neither received any immunization nor challenged. Data represent the OD_450 nm_ of at least three to five chicken samples in triplicate for IFNγ titers and three or four spleen chicken for cell proliferation assay in triplicate. Data in **B** were normalized with the corresponding data in the Neg. Ctrl. group. Data were normalized with the Neg. Ctrl. group in each time points. The bold font in **B** represents the statistically significant *p* values (*p* < 0.05).

The induced cell-mediated immunity was detected by quantifying the IFN-γ levels in the chicken’s serum. The significant increase in the IFN-γ levels showed that the BGs and killed vaccines could induce cellular immunity after two and three-times vaccination at days 21 and 28 (*p* < 0.05). After three times vaccination, the IFN-γ levels increased at day 28 while the production peak was observed after challenge with the wild type O78:K80 bacteria at day 38 (Figure 3A, B and Additional file 6). Birds in the vaccinated groups showed a peak of IFN-γ levels, which was significantly higher than that in non-immunized groups (*p* < 0.05). The *p* value comparison analysis of IFN-γ titers (normalized with the titers of the Neg. Ctrl group) in the immunized and exposed groups on days 28 and 38 was significantly higher than those of day 14 (Figure 3B and Additional file 6).

To evaluate the cell-mediated immune responses, the lymphocyte activation and proliferation were measured by analyzing lymphocytes isolated from the spleen of immunized and non-immunized birds at days 21, 28, and 38. The immunized birds in Inj. BGs + Expo. and killed + Expo. groups showed significantly higher proliferation than the non-immunized birds in the Neg. Ctrl. and only Expo. groups at day 21 (*p* < 0.05). There were no significant differences between groups on day 28, but on day 38, the Inhal. BGs + Expo and killed + Expo groups had a significantly higher percentage of proliferative lymphocytes than the Neg. Ctrl. group (*p* < 0.05) (Figure 3C).

### Expression profile of proinflammatory cytokines

**Figure 4:**
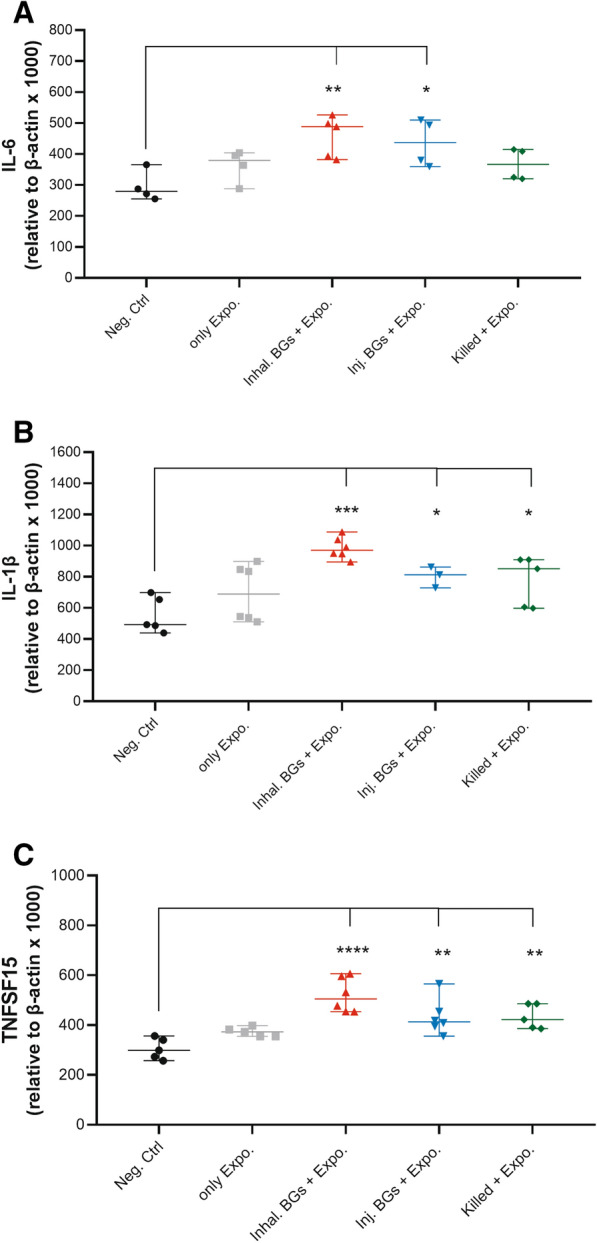
**Expression profile of proinflammatory cytokines.** RT-qPCR analysis of proinflammatory cytokines including IL-6 (**A**), IL-1β (**B**), and TNFSF15 (**C**) in the immunized and non-immunized chickens at days 38, after challenging with the wild type *E. coli* O78:K80. The transcripts for each gene were quantified relative to β-actin levels in each group. The absolute copy number for IL-6, IL-1β, TNFSF15, and β-actin transcripts were quantified based on the related standard curves, and for three series of cDNAs from chicken PBMCs. The significant differences between groups are shown as ****p ≤ 0.0001, ***p ≤ 0.001, **p ≤ 0.01 and *p ≤ 0.05. Black dots: negative control group; grey squares: only Exposure group; red triangles: inhalation. BGs + Exposure group; blue triangle: injection. BGs + Exposure group; green squares: killed vaccine + Exposure group.

To determine the expression of inflammatory cytokines in PBMCs after three times of immunization and after challenge, quantitative real-time PCR was performed. Expression of IL-6 was significantly upregulated in the two BG-vaccinated groups indicating that BGs could induce the level of cytokine transcripts compared to the Neg. Ctrl. group (*p* < 0.05). Birds vaccinated with the killed vaccine showed increased expression of IL-6 compared to the untreated birds in the Neg. Ctrl. group although the difference was not significant (Figure 4A). The results of the expression level of IL-1β and TNFSF15 demonstrated that the vaccine administration leads to a significant increase in cytokines production compared with the Neg. Ctrl group (*p* < 0.05) (Figures 4B, C). Analysis of the results of candidate cytokine copy numbers in this study showed that BG-vaccinated birds through inhalation had the highest cytokine expression compared to the other vaccinated groups but there were no significant differences between vaccinated groups (*p* > 0.05) (Figure 4A–C).

### Necroscopy findings

**Figure 5:**
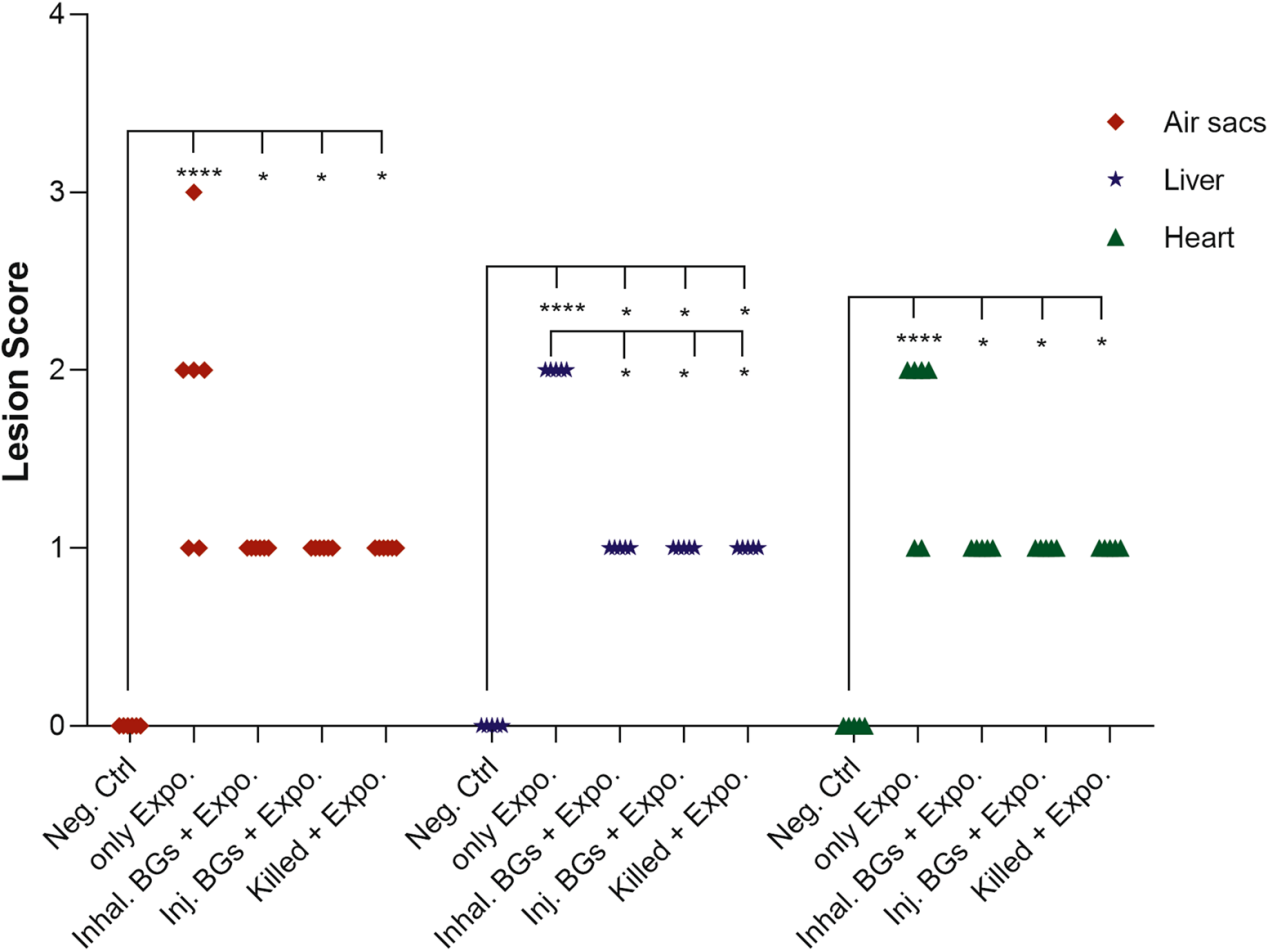
**Lesion scoring in immunized and non-immunized groups of chicken after challenge with the wild type** ***E. coli*** **O78:K80.** The lesion scoring for every carcass was performed for air sacs, heart, and liver as described in “Materials and methods” section. The statistically significant differences between groups are shown as ****p ≤ 0.0001 and *p ≤ 0.05.

After a challenge with the wild type O78:K80 bacteria, the organ lesions were scored in different groups of chicken. In the vaccinated groups, the air sacs, liver, and heart had lower lesion scores in comparison with the only Expo. group. No lesions were observed in the Neg. Ctrl. group; therefore, the organ scores were 0 and there was a significant difference between this group and the only Expo. and three immunized groups (*p* < 0.05). The lesion scores in the liver were significantly different between the three vaccinated and the only Expo. groups. There were no significant differences between the killed vaccines and the two types of BG vaccines (Figure 5).

## Discussion

In this study, we have designed and developed a candidate nucleic-acid free bacterial ghost vaccine against APEC, O78:K80 serotype. Vaccination plays an important role in the health management of the poultry industry [33]. There are numerous bacterial infectious diseases, such as APEC causing colibacillosis, which could be prevented by vaccination. An ideal vaccine for APEC has to overcome several challenges, for example, to induce cross-protection against various APEC serogroups involved in the disease; could be deliverable through a mass immunization method such as through the drinking water or feed, in ovo, and spraying; and finally could be administered to young-aged chickens to develop a whole protective immunity before day 21 when they are most susceptible to APEC [2, 12].

To achieve the complete inactivation of the host bacteria and removal of genomic content from O78:K80 bacterial cells, the expression of the SNUC gene along with E-Lysis was induced, leading to no detectable viable cells at the end of BGs production procedure and no detection of genomic and plasmid DNA. Therefore, the complete inactivation of any remaining DNA has minimized the risk of introducing antibiotic resistance genes or pathogenic islands to hosts [17, 25]. The functionality of SNUC protein was confirmed by gel electrophoresis of genomic DNA and absolute qPCR for the detection of pmET32c plasmid (Figure 1D–F). These findings were confirmed by SEM imaging for representing the pores in the bacterial cell wall (Figure 1B), the drop of optical density indicating bacterial growth dynamics, and decreased colony number count during BGs generation (Figure 1C and Additional file 5). Therefore, these O78:K80 BGs subsequently were evaluated as a potential vaccine against chicken colibacillosis. It has previously been reported that the relevant immunogenic epitopes on these bacterial cell surfaces preserve their conformational structure, which is necessary for the efficient stimulation of the immune system [25].

Analysis of immune responses in chickens immunized with O78:K80 BGs indicated that the immunized birds have developed protective responses. The protection depends on the elicitation of the immune responses through innate and adaptive immunity. The findings of different studies reviewed by Hajam et al. [25] suggest that BG vaccines can stimulate both humoral and cell-mediated immune responses. It is believed that serogroup-specific protection against autologous bacteria acts principally through the induction of humoral responses. The serogroup-specificity of such vaccines has been inferred to be due to the dominance of responses to the lipopolysaccharide O antigen [34]. On the other hand, the presence of LPS in BG might have the ability to stimulate B cells directly through the TLR4 pathway and subsequently help in the generation of potent antibody formation [25]. Overall, it seems that for the protection of chickens against O78K80 bacteria using O78:K80 BGs, besides the expression of the proinflammatory cytokines, the most important contributor to immunity is the significantly high production of humoral antibody responses (IgY and IgA), followed by T cell responses. The O78:K80 BGs elicit the increased expression of the proinflammatory cytokines, IL-6, IL-1β, and TNFα (TNFSF15), which could be efficient for adaptive immune responses (Figure 4A–C). The O78:K80 BGs also induced more potent IL-6 expression than the killed vaccine (Figure 4A). These cytokines can activate adaptive immune responses. The expression of these cytokines results in the enhanced activation of B and T cells leading to the stimulation of efficient humoral and cellular immune responses. The birds immunized with O78:K80 BGs and exposed to wild-type bacteria by inhalation and injection routes generated high titers of IgY and IgA antibodies (Figure 2A, C). By increasing the number of vaccinations, these antibodies showed higher titers (Figure 2B, D). The high level of IFNɣ (Figure 3A, B), along with IL-6, IL-1β, and TNFα (TNFSF15), can elicit a stronger Th1 cellular response [14, 35]. Immune cellular responses for the candidate vaccine caused an increased proliferation of spleen lymphocytes (Figure 3C). In the vaccinated birds, lesions caused by the wild-type bacteria were reduced in the liver, heart, and air sacs in comparison to non-vaccinated controls (Figure 5).

**Figure 6:**
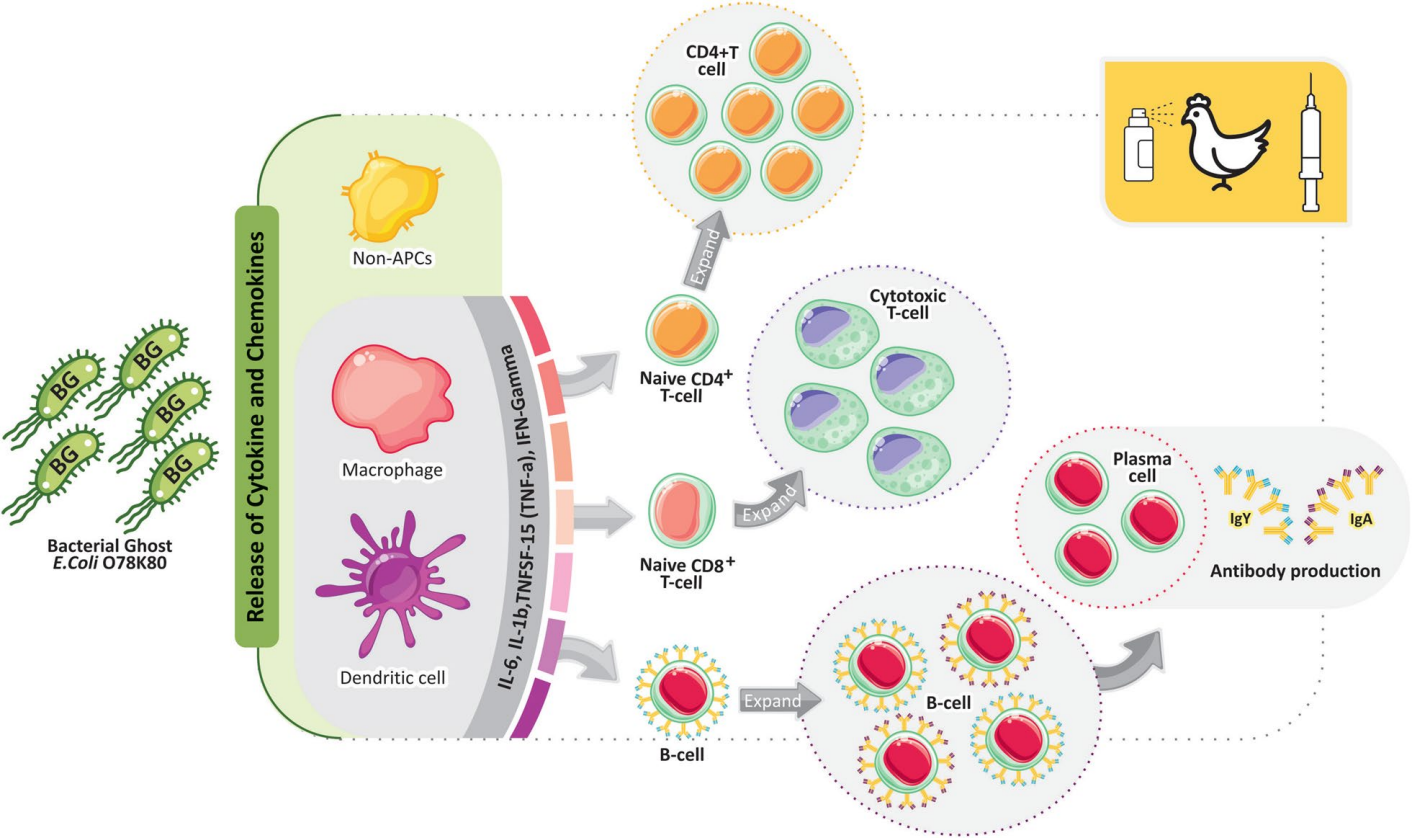
**Schematic representation of host chickens immune response to vaccination with a nucleic acid-free version of** ***E. coli*** **O78:K80 BGs.** The O78:K80 BG vaccine candidate, used through injection and inhalation routes, triggered higher proinflammatory cytokine expression including IL-6, IL-1β, and TNFSF15; a higher level of antibody-dependent humoral (IgY and IgA) and cellular immune responses (IFNγ and lymphocyte proliferation). Accordingly, it seems that the O78:K80 BGs have the potential to recruit innate and adaptive immune cells to generate antibodies and T cell responses and protection against the challenge.

The results of this study indicate that O78:K80 BGs have the potential to recruit innate and adaptive immune cells to generate antibodies and T cell responses (Figure 6). Since the respiratory system is known as the route of entry for *E. coli* in poultry colibacillosis [10], it is desirable to develop vaccines that can induce antibody responses in the respiratory tract of the chickens. The antibodies from the mucosal secretions of the respiratory tract can prevent infection and spread of the disease [15]. Also, it is demonstrated that IL-6 cytokine helps in the development of effective mucosal immune responses [25]. Our observations in this study indicated that the O78:K80 BGs that were delivered by spray have the potential to induce a potent mucosal immune response, comparable with the injectional route. Administration of mucosal vaccines is an attractive idea since the BG vaccines with their intrinsic surface adjuvant molecules can induce efficient systematic and mucosal immunity to intensify immune responses against wild-type bacterial surface antigens.

Several publications have tested *E. coli* BGs as an empty cell envelope in different animal models. We reviewed the *E. coli* strains, methodology, and results of five related publications [26–28, 36, 37] to ours (Table 2). Mayr and colleagues [26, 37] tested EHEC O157H7 BGs against the lethal challenge of O157H7 in mice through the oral and rectal routes. The results of these studies showed that the O157H7 BGs induced potent humoral and cellular immune responses and fully protected the mice against the EHEC strain. Jiangang Hu et al. evaluated the immunological effects of the BG vaccine against the luxS-aroA double-gene deleted APEC mutant (strain DE17ΔluxSΔaroA, O2 serotype). The results showed that the BG vaccine was able to achieve over 90% immune protection against virulent challenge using the same serotype O2 strain (DE17 or CE35), while it showed poor cross-protection against serotypes O1 and O78 [36]. In our research group, there have been two publications for evaluation of the efficacy of O78:K80 and O2K1 BGs against the homologous strains in chicken [27, 28]. As shown in Table 2, in these studies, BGs production was performed only by the expression of E-lysis protein. The results of the efficacy for these candidate vaccines demonstrated their potential to elicit immune responses and protection against the challenge. In this study, we challenged the vaccinated birds with higher doses than previously used. Our findings demonstrated that the immune responses were very close to that of the killed vaccine. This implies that our nucleic-acid free BG candidate vaccine provides higher efficacy and safety in comparison to what has been previously reported.

**Table 2:** **Comparison of methodology and results of research reports analyzing** ***E.coli*** **BGs as empty cell envelope**

Candidate vaccine [Refs.]	BGs production	Model/route/BGs dose	Assayed factor/results
EHEC O157H7 [26]	E-Lysis and SNUC	Mice/oral/4.8 × 10^9^ BGs	IgG, IgA, IFNγ, cell proliferation/humoral and CMI response, protection against lethal heterologous challenge
EHEC O157H7 [37]	E-Lysis and SNUC	Mice/rectal/4.8 × 10^9^ BGs	IgG, IgA, IFNγ, cell proliferation/humoral and CMI response, protection against lethal heterologous challenge
APEC O2 (DE17ΔluxSΔaroA) [36]	E-Lysis and SNUC	Chicken/S.C./10^9^ CFU/0.3 mL	Histopathological analysis, IgG, IFNγ, TNF-α, IL-6/o pathological changes, IgG, IFNγ and TNF-α production
APEC O2K1 [28]	E-Lysis	Chicken/Inha., Inj./10^10^ CFU/mL	IgY, IgA, IFNγ, lesion scores/IgY, IgA antibodies and IFNγ production, reduced lesions in internal organs
APEC O78K80 [27]	E-Lysis	Chicken/Inha., Inj./10^10^ BGs/mL	IgY, IgA, IFNγ, lesion scores/IgY, IgA antibodies and IFNγ production, reduced lesions in internal organs
APEC O78K80 (this study)	E-Lysis and SNUC	Chicken/Inha., Inj./10^11^ BGs/mL	IgY, IgA, IFNγ, cell proliferation, proinflammatory cytokines expression, lesion scores/humoral and CMI response, IL-6, IL-1β, and TNFα (TNFSF15) expression, reduced lesions in internal organs

*S.C.* subcutaneous, *Inha.* inhalation, *Inj.* injection, *CMI* cell mediated immunity.

In conclusion, we have designed and developed a new nucleic-acid free candidate vaccine for colibacillosis, caused by the O78:K80 serotype, using bacterial ghost technology. Our results indicated that this candidate vaccine has protection potential against a homologous challenge to prevent the consequences of colibacillosis in chickens, comparable with the killed vaccine. This technology could also be applied to develop a multivalent vaccine to induce proper immunity against more prevalent APEC strains.

## Supplementary information

**Additional file 1.** **Construction of pmET32c plasmid for bacterial ghost production.** Subcloning of the SNUC gene into the pmET32b vector was performed to generate the pmET32c vector. The procedure was confirmed with colony PCR (A) and enzymatic digestion (B) which is visualized by gel electrophoresis. The product size for colony PCR using SNUC-F and SNUC-R was 513 bp. In single and double enzymatic digestions, the construct showed correct bands. Lane (a) shows uncut pmET32c vector. The single digestion of the construct with BglI or SalI generated a linear 7425 bp vector (b, c). The double digestion of construct with BglI and SalI generated two bands of 5328 and 2097 bp (d). Features of the pmET32c vector are shown in (C).

**Additional file 2.** Setup of qPCR analysis using the pmET32c vector. Location of designed quantitative-PCR primers in the pmET32c vector shown as pmET32c-F and pmET32c-R (A), Agarose gel electrophoresis of PCR amplicon using these primers (B), and melt curve analysis of the amplicon (C).

**Additional file 3.** Amplification and standard curves for RT-qPCR of different genes. The schematic representation of designed real-time PCR primers in the chicken genome, agarose gel electrophoresis of PCR amplicons, and standard and amplification curves for ACTB, IL-1b, IL-6, and TNFSF-15 genes.

**Additional file 4.** Representative images for lesion scoring of air sac, heart, and liver of carcasses. The details for the scoring index were described in the Material and methods (“Evaluation of necropsy lesions in liver, heart, and air sacs” section).

**Additional file 5.** Colony count at different time points of the BG production procedure. The Bacterial colonies were counted at different time points of the BG production procedure. The samples were diluted in 0.9% NaCl, and 100 µL of the 10^–3^ dilution was cultured on each LB agar plate and cultured O/N at 28 °C.

**Additional file 6.** ELISA titers of IgY, IgA, and IFNγ in 14-, 21-, 28-, and 38-day old chickens. Titers of IgY for four groups of chicken, just exposed (Expo.) (A1), Inhal. BGs + Expo. (A2), Inj. BGs + Expo. (A3), and Killed + Expo. (A4). Titers of IgA for four groups of chicken, just exposed (Expo.) (B1), Inhal. BGs + Expo. (B2), Inj. BGs + Expo. (B3), and Killed + Expo. (B4). Titers of IFNγ for four groups of chicken, just exposed (Expo.) (C1), Inhal. BGs + Expo. (C2), Inj. BGs + Expo. (C3), and Killed + Expo. (C4). Values are normalized with Neg. Ctrl average at different time points. The *p* values for statistically significant groups are shown. Chicken samples taken at different ages are shown with grey square (14-day old), red triangle (21-day old), blue triangle (28-day old), and green triangle (38-day old).

## Data Availability

The datasets supporting the conclusions of this article are included within the article (and its additional files) and also are available from the corresponding author upon request.

## Abbreviations

APEC: avian pathogenic *Escherichia coli*
BG: bacterial ghost
SNUC: staphylococcal nuclease or S nuclease
IL-6: interleukin-6
IL-1β: interleukin-1beta
TNFSF15: tumor necrosis factor superfamily member 15
IFNγ: interferon-gamma
SEM: scanning electron microscopy
ELISA: indirect enzyme-linked immunosorbent assay
PBMCs: peripheral blood mononuclear cells
AB: AlamarBlue
