# Supplementary material for: Design, development, and evaluation of the efficacy of a nucleic acid-free version of a bacterial ghost candidate vaccine against avian pathogenic E. coli (APEC) O78:K80 serotype
Source: Vet Res. 2020 Dec 9;51:144. doi: 10.1186/s13567-020-00867-w (PMC7724879; doi:10.1186/s13567-020-00867-w)
Supplement: Supplementary file 1 — Additional file 1. Construction of pmET32c plasmid for bacterial ghost production. Subcloning of the SNUC gene into the pmET32b vector was performed to generate the pmET32c vector. The procedure was confirmed with colony PCR (A) and enzymatic digestion (B) which is visualized by gel electrophoresis. The product size for colony PCR using SNUC-F and SNUC-R was 513 bp. In single and double enzymatic digestions, the construct showed correct bands. Lane (a) shows uncut pmET32c vector. The single digestion of the construct with BglI or SalI generated a linear 7425 bp vector (b, c). The double digestion of construct with BglI and SalI generated two bands of 5328 and 2097 bp (d). Features of the pmET32c vector are shown in (C). [file 13567_2020_867_MOESM1_ESM.docx]

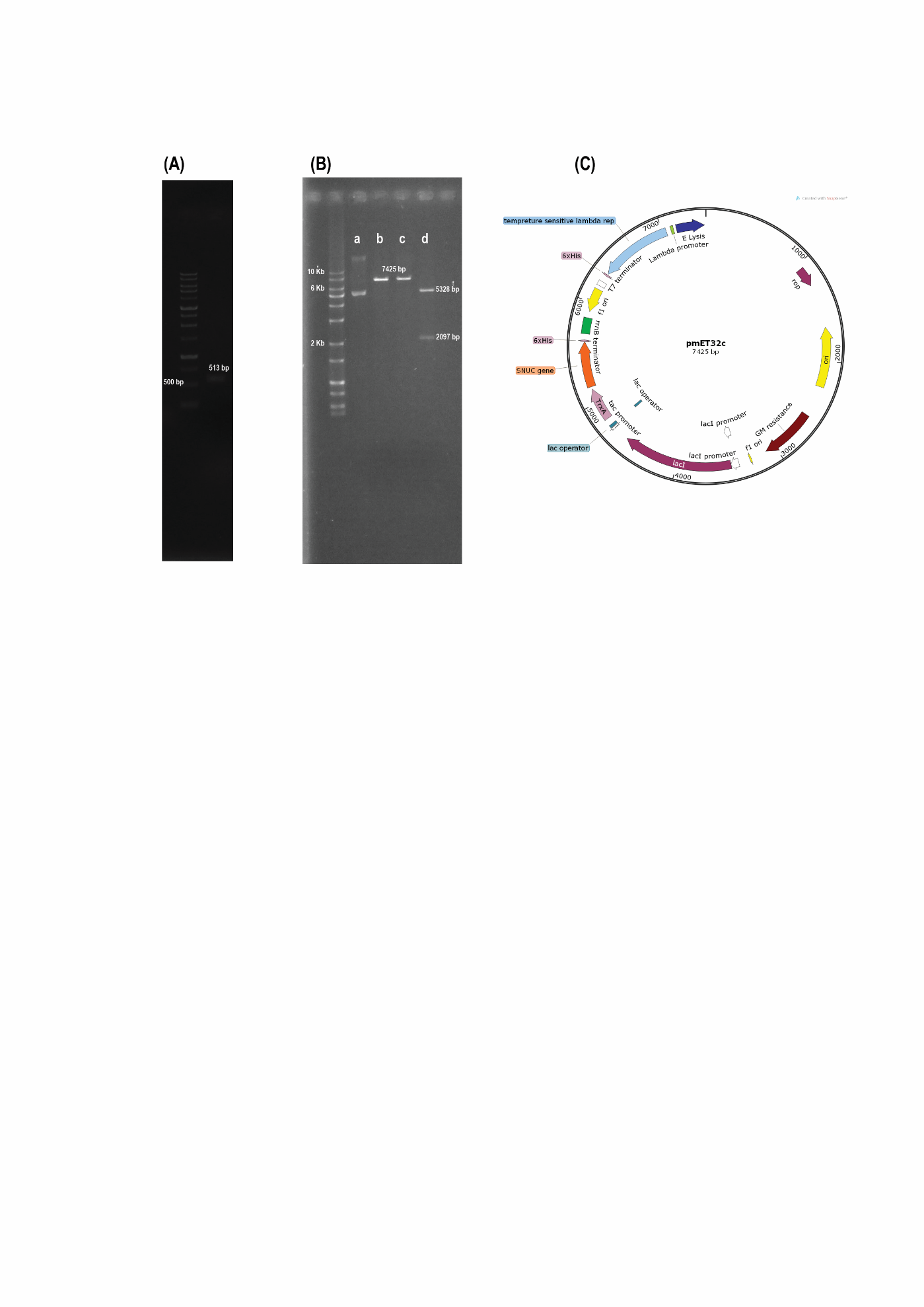


**Additional file 1. Construction of pmET32c plasmid for bacterial ghost production.** Subcloning of the SNUC gene into the pmET32b vector was performed to generate the pmET32c vector. The procedure was confirmed with colony PCR (A) and enzymatic digestion (B) which is visualized by gel electrophoresis. The product size for colony PCR using SNUC-F and SNUC-R was 513 bp. In single and double enzymatic digestions, the construct showed correct bands. Lane (a) shows uncut pmET32c vector. The single digestion of the construct with BglI or SalI generated a linear 7425 bp vector (b, c). The double digestion of construct with BglI and SalI generated two bands of 5328 and 2097 bp (d). Features of the pmET32c vector are shown in (C).
