# Supplementary material for: Design, development, and evaluation of the efficacy of a nucleic acid-free version of a bacterial ghost candidate vaccine against avian pathogenic E. coli (APEC) O78:K80 serotype
Source: Vet Res. 2020 Dec 9;51:144. doi: 10.1186/s13567-020-00867-w (PMC7724879; doi:10.1186/s13567-020-00867-w)
Supplement: Supplementary file 2 — Additional file 2. Setup of qPCR analysis using the pmET32c vector. Location of designed quantitative-PCR primers in the pmET32c vector shown as pmET32c-F and pmET32c-R (A), Agarose gel electrophoresis of PCR amplicon using these primers (B), and melt curve analysis of the amplicon (C). [file 13567_2020_867_MOESM2_ESM.docx]

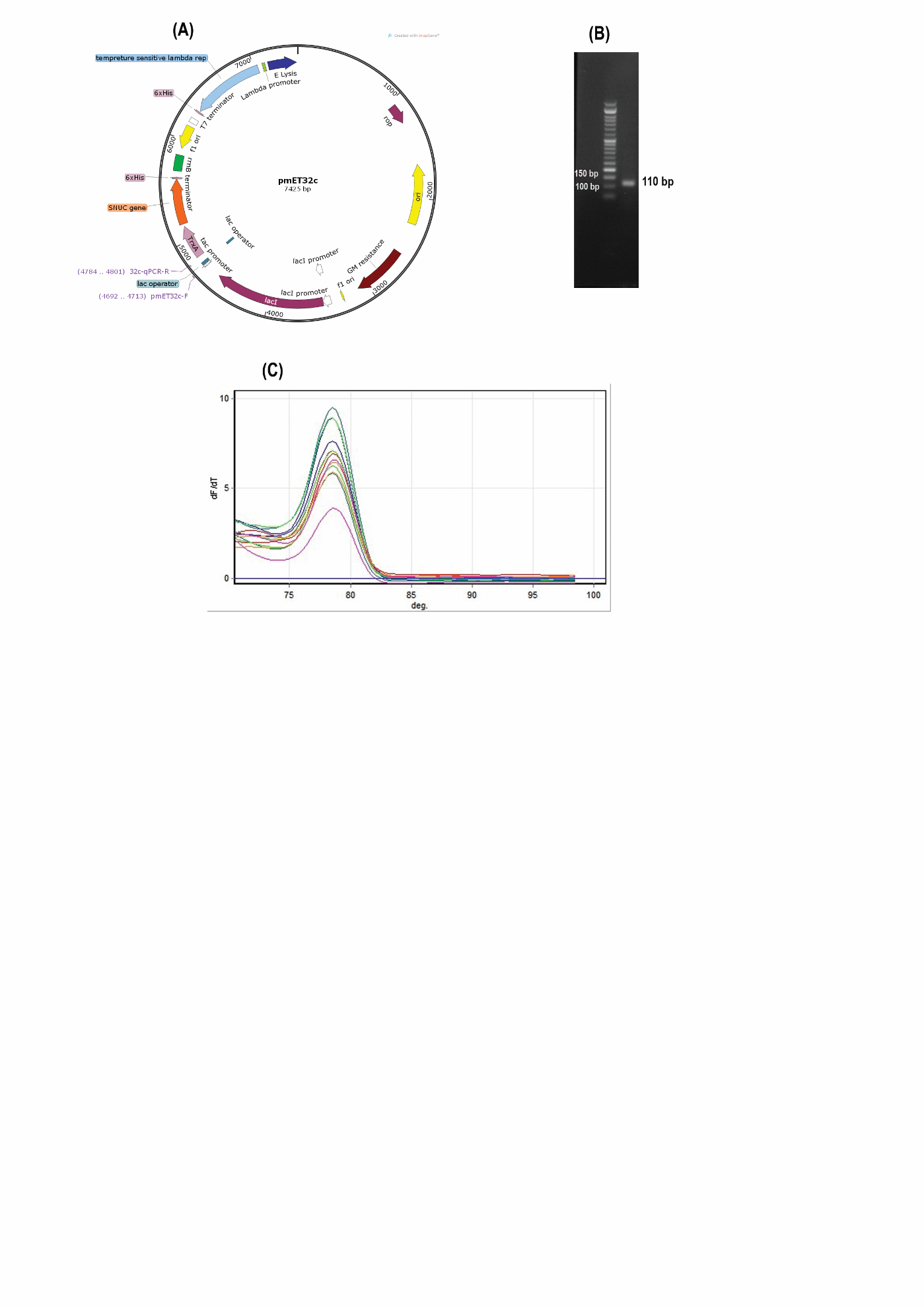


**Additional file 2. Setup of qPCR analysis using the pmET32c vector.** Location of designed quantitative-PCR primers in the pmET32c vector shown as pmET32c-F and pmET32c-R (**A**), Agarose gel electrophoresis of PCR amplicon using these primers (**B**), and melt curve analysis of the amplicon (**C**).
