# Supplementary material for: Design, development, and evaluation of the efficacy of a nucleic acid-free version of a bacterial ghost candidate vaccine against avian pathogenic E. coli (APEC) O78:K80 serotype
Source: Vet Res. 2020 Dec 9;51:144. doi: 10.1186/s13567-020-00867-w (PMC7724879; doi:10.1186/s13567-020-00867-w)
Supplement: Supplementary file 3 — Additional file 3. Amplification and standard curves for RT-qPCR of different genes. The schematic representation of designed real-time PCR primers in the chicken genome, agarose gel electrophoresis of PCR amplicons, and standard and amplification curves for ACTB, IL-1b, IL-6, and TNFSF-15 genes. [file 13567_2020_867_MOESM3_ESM.docx]

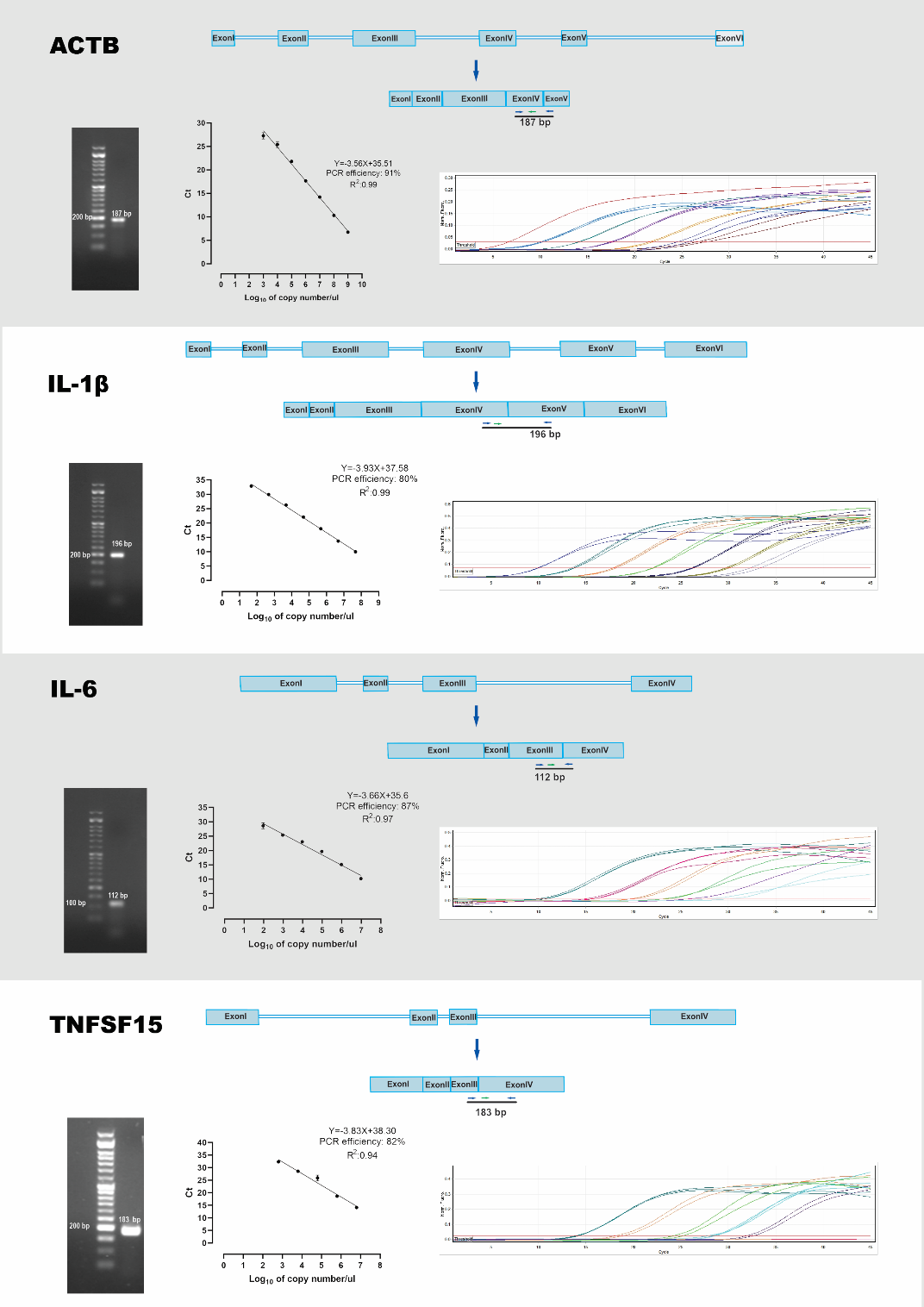


**Additional file 3. Amplification and standard curves for RT-qPCR of different genes.** The schematic representation of designed real-time PCR primers in the chicken genome, agarose gel electrophoresis of PCR amplicons, and standard and amplification curves for ACTB, IL-1b, IL-6, and TNFSF-15 genes.
