# Supplementary material for: Design, development, and evaluation of the efficacy of a nucleic acid-free version of a bacterial ghost candidate vaccine against avian pathogenic E. coli (APEC) O78:K80 serotype
Source: Vet Res. 2020 Dec 9;51:144. doi: 10.1186/s13567-020-00867-w (PMC7724879; doi:10.1186/s13567-020-00867-w)
Supplement: Supplementary file 4 — Additional file 4. Representative images for lesion scoring of air sac, heart, and liver of carcasses. The details for the scoring index were described in the Material and methods (“Evaluation of necropsy lesions in liver, heart, and air sacs” section). [file 13567_2020_867_MOESM4_ESM.docx]

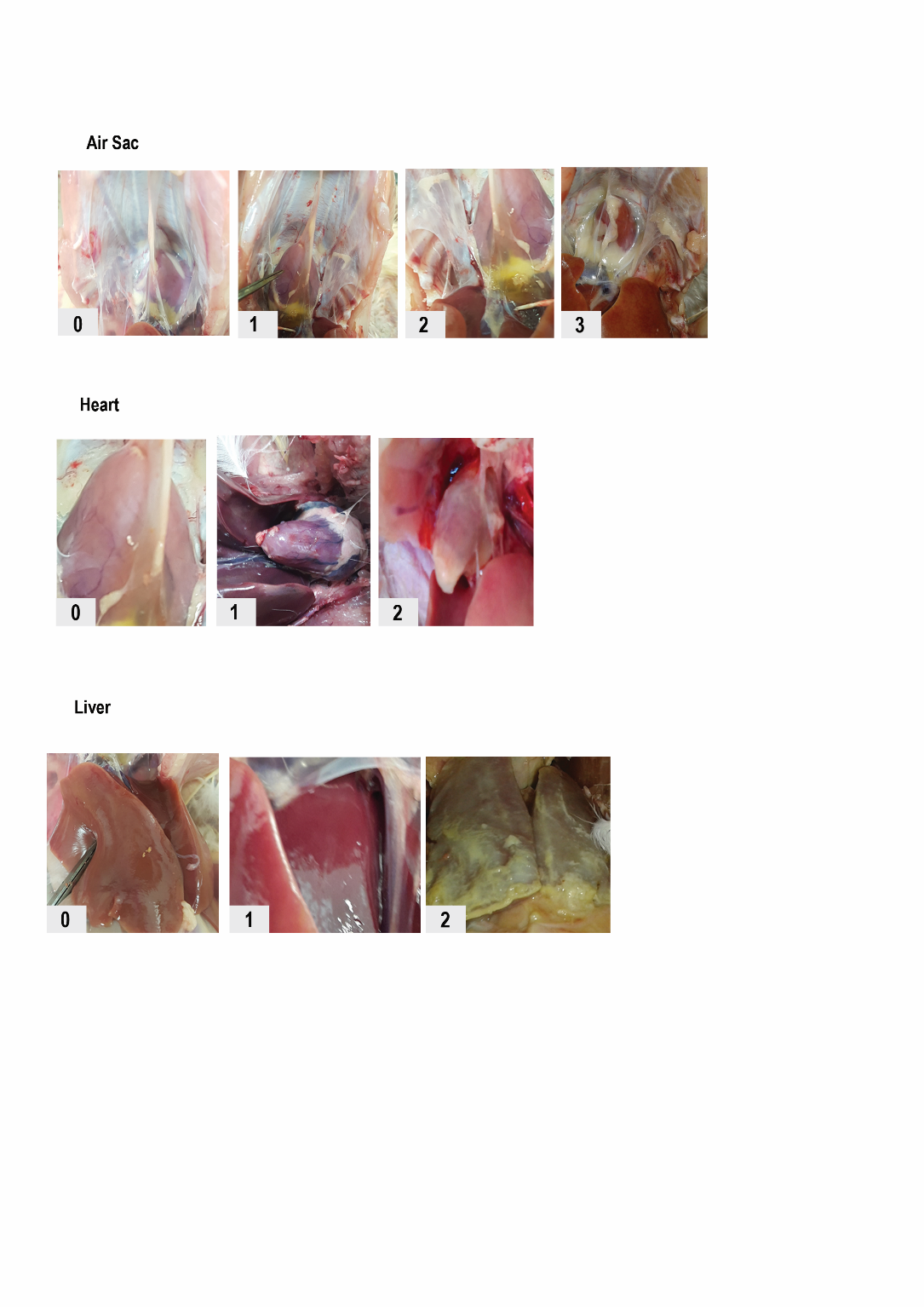


**Additional file 4. Representative images for lesion scoring of air sac, heart, and liver of carcasses.** The details for the scoring index were described in the Material and methods (section 2.5).
