# Supplementary material for: Design, development, and evaluation of the efficacy of a nucleic acid-free version of a bacterial ghost candidate vaccine against avian pathogenic E. coli (APEC) O78:K80 serotype
Source: Vet Res. 2020 Dec 9;51:144. doi: 10.1186/s13567-020-00867-w (PMC7724879; doi:10.1186/s13567-020-00867-w)
Supplement: Supplementary file 5 — Additional file 5. Colony count at different time points of the BG production procedure. The Bacterial colonies were counted at different time points of the BG production procedure. The samples were diluted in 0.9% NaCl, and 100 µL of the 10–3 dilution was cultured on each LB agar plate and cultured O/N at 28 °C. [file 13567_2020_867_MOESM5_ESM.docx]

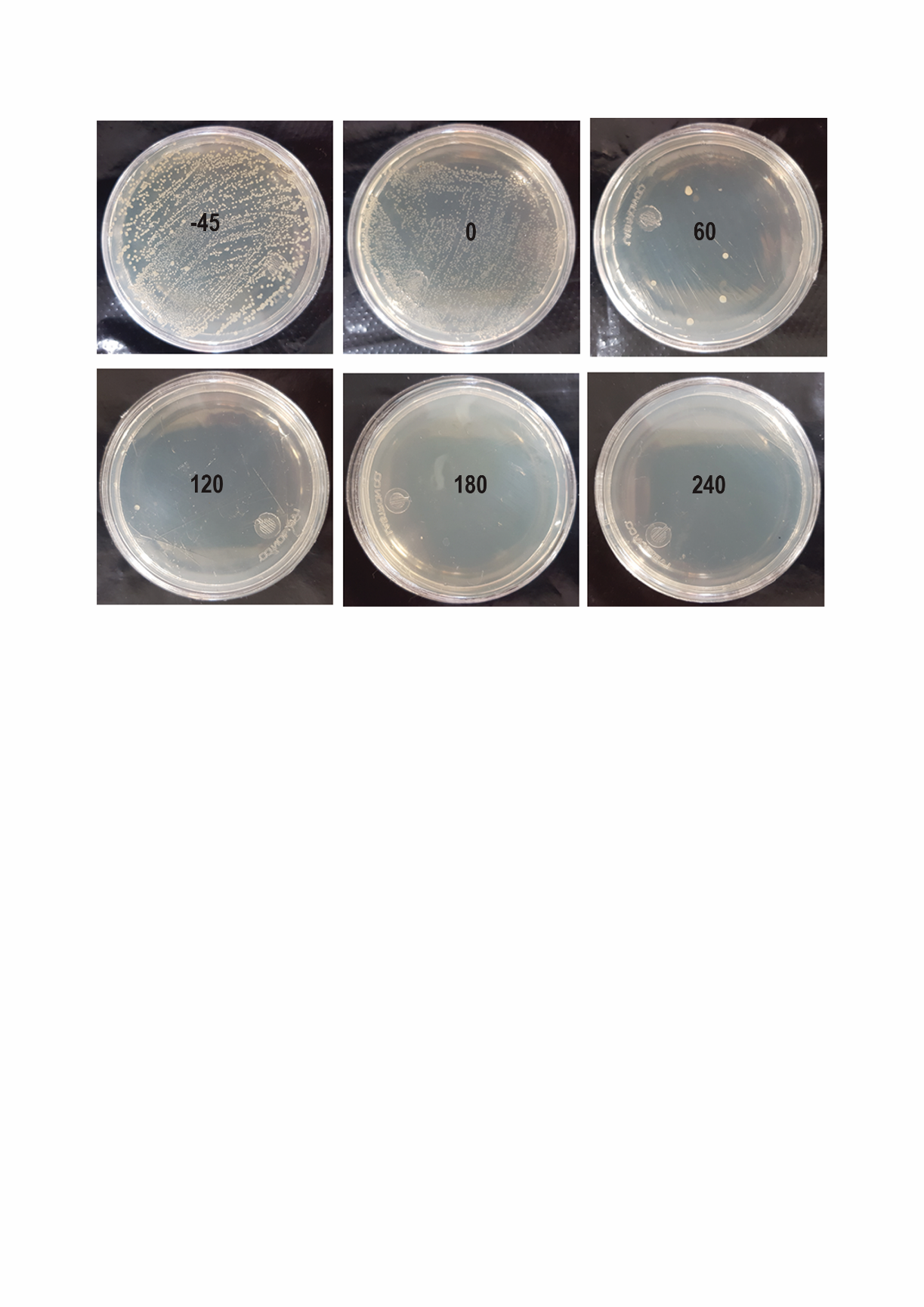


**Additional file 5. Colony count at different timepoints of BG production procedure.** The Bacterial colonies were counted at different time points of BG production procedure. The samples were diluted in 0.9 % NaCl, and 100 µl of the 10^-3^ dilution was cultured on each LB agar plate, and cultured O/N at 28 °C.
