# Supplementary material for: Design, development, and evaluation of the efficacy of a nucleic acid-free version of a bacterial ghost candidate vaccine against avian pathogenic E. coli (APEC) O78:K80 serotype
Source: Vet Res. 2020 Dec 9;51:144. doi: 10.1186/s13567-020-00867-w (PMC7724879; doi:10.1186/s13567-020-00867-w)
Supplement: Supplementary file 6 — Additional file 6. ELISA titers of IgY, IgA, and IFNγ in 14-, 21-, 28-, and 38-day old chickens. Titers of IgY for four groups of chicken, just exposed (Expo.) (A1), Inhal. BGs + Expo. (A2), Inj. BGs + Expo. (A3), and Killed + Expo. (A4). Titers of IgA for four groups of chicken, just exposed (Expo.) (B1), Inhal. BGs + Expo. (B2), Inj. BGs + Expo. (B3), and Killed + Expo. (B4). Titers of IFNγ for four groups of chicken, just exposed (Expo.) (C1), Inhal. BGs + Expo. (C2), Inj. BGs + Expo. (C3), and Killed + Expo. (C4). Values are normalized with Neg. Ctrl average at different time points. The p values for statistically significant groups are shown. Chicken samples taken at different ages are shown with grey square (14-day old), red triangle (21-day old), blue triangle (28-day old), and green triangle (38-day old). [file 13567_2020_867_MOESM6_ESM.docx]

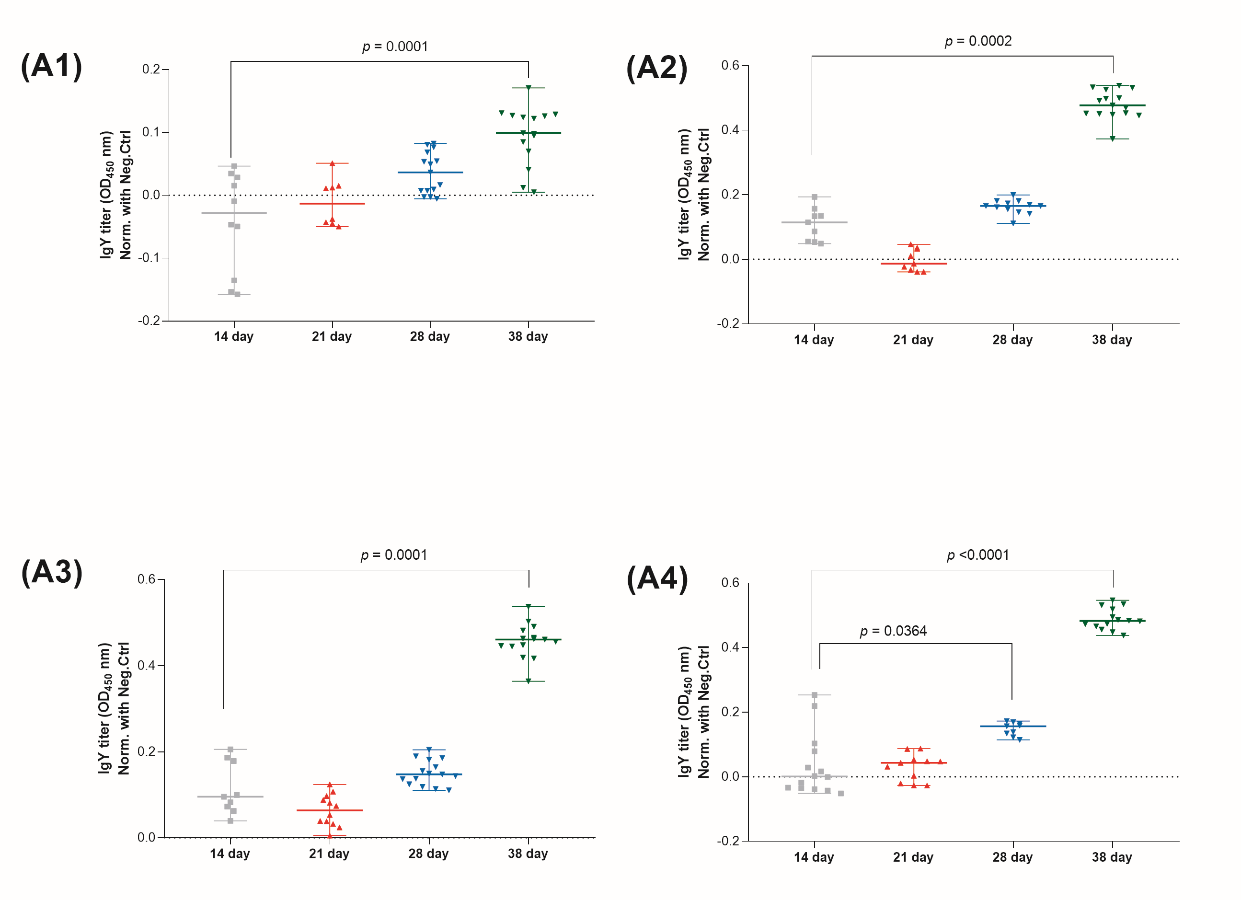


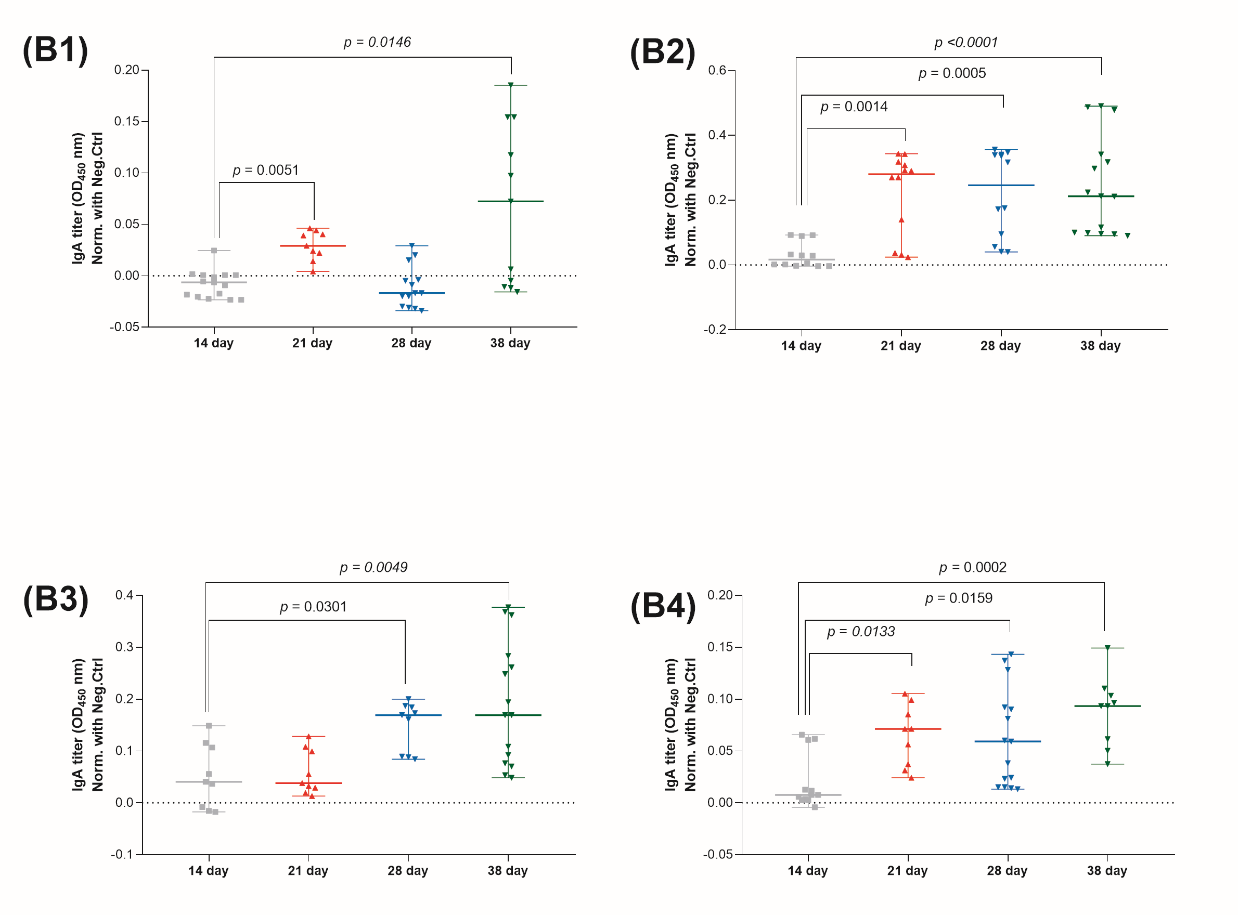


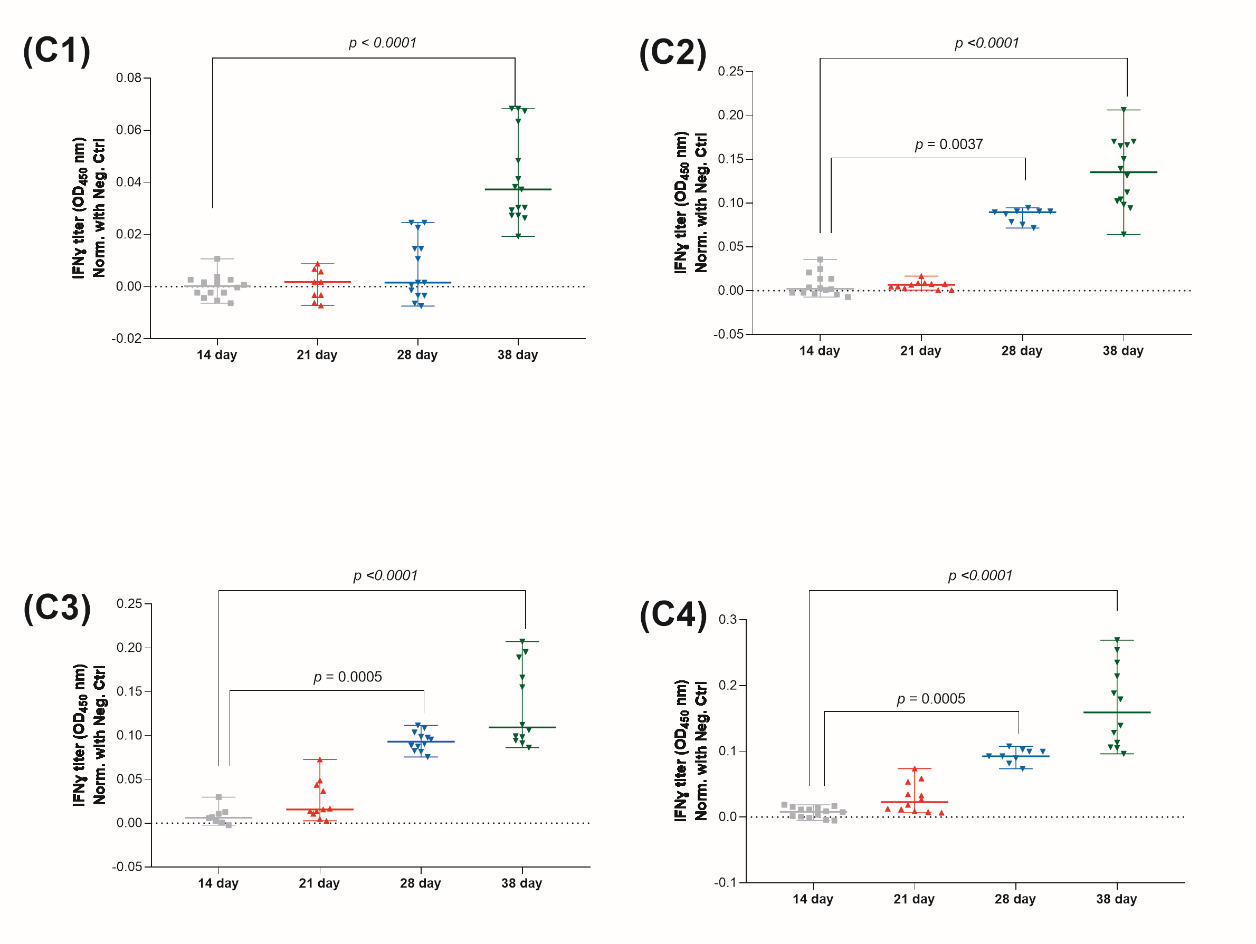


**Additional file 6. ELISA titers of IgY, IgA, and IFNγ in 14-, 21-, 28-, and 38-day old chickens.** Titers of IgY for four groups of chicken, just exposed (Expo.) (A1), Inhal. BGs + Expo. (A2), Inj. BGs + Expo. (A3), and Killed + Expo. (A4). Titers of IgA for four groups of chicken, just exposed (Expo.) (B1), Inhal. BGs + Expo. (B2), Inj. BGs + Expo. (B3), and Killed + Expo. (B4). Titers of IFNγ for four groups of chicken, just exposed (Expo.) (C1), Inhal. BGs + Expo. (C2), Inj. BGs + Expo. (C3), and Killed + Expo. (C4). Values are normalized with Neg. Ctrl average at different time points. The *p*-values for statistically significant groups are shown. Chicken samples taken at different ages are shown with grey square (14-day old), red triangle (21-day old), blue triangle (28-day old), and green triangle (38-day old).
